# Aggregation of alpha-synuclein in enteric neurons does not impact function in vitro

**DOI:** 10.1038/s41598-022-26543-x

**Published:** 2022-12-23

**Authors:** Adam J. Bindas, Kyla N. Nichols, Nicole J. Roth, Ryan Brady, Abigail N. Koppes, Ryan A. Koppes

**Affiliations:** 1 Department of Chemical Engineering, Northeastern University, 360 Huntington Ave., 339 Mugar, Boston, MA 02115 USA; 2 Department of Biology, Northeastern University, 360 Huntington Ave., 340 Mugar, Boston, MA 02115 USA; 3 Geriatrics Section, Department of Medicine, Boston Medical Center, One Boston Medical Center Pl., Boston, MA 02118 USA

**Keywords:** Parkinson's disease, Intrinsically disordered proteins

## Abstract

Recent evidence implicates a gut-first pathogenesis in the enteric nervous system (ENS) within a portion of PD patients, yet in vitro investigations have primarily focused on the central nervous system. Here, the preformed fibril (PFF) PD model is applied with co-administered groups of butyrate and lipopolysaccharide to model the effects of the local gut microbiome. Significant PFF uptake and retention occur in isolated rat enteric neurons compared to untreated controls resulting in increasing immunostained aggregate conformation-specific, alpha-synuclein (a-Syn) average intensity between 6 µg PFF and untreated controls. Cortical neurons significantly retain PFFs with an increase in aggregated a-Syn average intensity within all dosages. Differences in growth cone morphology but not dynamics in PFF-treated ENS cultures occur. Electrophysiological recordings via a microelectrode array (MEA) indicate no overall difference in spontaneous spike rate. However, only untreated controls respond to PD-relevant dopamine stimulus, while 1 µg PFF and control populations respond to stimulus with ENS-abundant acetylcholine. Finally, no differences in substance P levels—correlated with PD and neurodegeneration—are observed. Overall, these findings suggest the ENS retains PFF dosage absent acute loss in function, however, does experience changes in growth cone morphology and dopamine-stimulated activity.

## Introduction

The enteric nervous system (ENS) has been implicated in hosting a “gut-first” etiology of Parkinson’s disease (PD)^1,2^. PD is defined by progressive mobility degradation and resting tremors from neural degeneration. Lewy pathology (LP), the neurological hallmark of the disease, can be classified by location: Lewy bodies are found within somas and Lewy neurites within extensions. LP is believed to form via aggregation of the plentiful α-synuclein (a-Syn) protein to oligomers and fibrils. While initial Parkinsonian symptoms are likely a result of dopamine deficiencies following neural loss in the substantia nigra, LP may originate in the periphery, then transport cell to cell to the central nervous system (CNS)^3–6^. Meta-analyses concluded that both symptomatic (gut dysfunction including constipation^7–9^) and pathological (Lewy pathology^10,11^) presentations occur in a portion of PD patients up to decades prior to the onset of cardinal motor symptoms. In addition, patients who undergo vagotomy have reduced PD risk^4,12^. These findings with vagotomy^3,13^ and also sympathectomy have been replicated in disease models, including with exogenously delivered preformed, a-Syn fibrils (PFFs)^3,13^, suggesting direct trafficking of fibrils from the gut to the CNS via both vagal and non-vagal pathways^3,13,14^. PFFs have been utilized for further research both in vitro and in vivo, demonstrating the capability of exogenous dosages to be endocytosed^15,16^, transported along axons bidirectionally, and propagated to neighboring cells^17^ inducing Parkinsonian-like motor dysfunction^3,18^ in mice and rats^1,14,19^.

Despite clinical and experimental evidence implicating the ENS in a subset of PD patients, limited in vitro investigations have been conducted on primary enteric neurons. Braidy et al. observed that enteric neurons endocytose PFFs, and cell death is initiated via decreased lysosomal capacity and mitochondrial complex I activity^15^. Pan-Montojo et al. observed supplemental rotenone resulted in increased a-Syn inclusion count and fluorescently-tagged a-Syn was retrogradely transported from enteric to sympathetic neurons^13^. Prigent et al. observed that primary rat ENS had decreased a-Syn expression in response to a lipopolysaccharide (LPS) and cytokine cocktail (tumor necrosis factor-alpha and interleukin 1-beta) via a P38 pathway^20^. Lassozé et al. compared the sensitivity and specificity of five commercially-available phosphorylated a-Syn antibodies (at Ser129) for aggregated a-Syn^21^. Finally, Gries et al., as part of a more extensive animal model assessment, demonstrated that primary enteric neurons dosed with aggregated A30P mutant a-Syn protein experienced a significant loss in neuronal viability, along with altered ratios of neurofilament light-chain and calbindin-2 positive neurons and vesicle-associated membrane protein 2^22^. These findings demonstrate the need for additional investigation of the impact of PFFs on enteric neurons as the cell response may differ from populations of the CNS.

Recent work has focused on environmental factors or stimuli that may initiate gut LP, implicating several cues, including pesticides and pathogenic bacteria^1,20,23–25^. Perez-Pardo et al. demonstrated that toll-like receptor 4 (TLR4) activation, which is induced by LPS, is associated with increased inflammation and degeneration in both the ENS and CNS^23^. While work has demonstrated the impacts of LPS on CNS LP formation in vitro, ENS and CNS populations have been shown to respond differently to LPS stimulus^20^. Thus, a gap exists in understanding how chronic LPS dosage impacts PFF-induced pathology in enteric neurons. Additionally, several studies have demonstrated the loss of butyrate and short-chain fatty acid (SCFA)-producing bacteria associated with PD^1,24,25^. Recently, Getachew et al. observed that the presentation of butyrate with toxic salsolinol, a product of dopamine metabolism found in the urine of PD patients, reduced cell death in an SH-SY5Y cell line^26^. Therefore, a need exists to assess the impact of butyrate on PFF and LPS toxicity. Here, we assess uptake and retention of fluorescently tagged PFFs by rat enteric neurons under different dosages and co-administer key luminal contents (butyrate and LPS) as modulators using live microscopy over a 21 days, after which the cultures are immunostained and analyzed for aggregated a-Syn average fluorescent intensity. Additionally, we assess morphological growth cone, electrophysiological firing, and substance P levels to infer changes in cellular function.

## Results and discussion

### Experimental design

**Figure 1:**
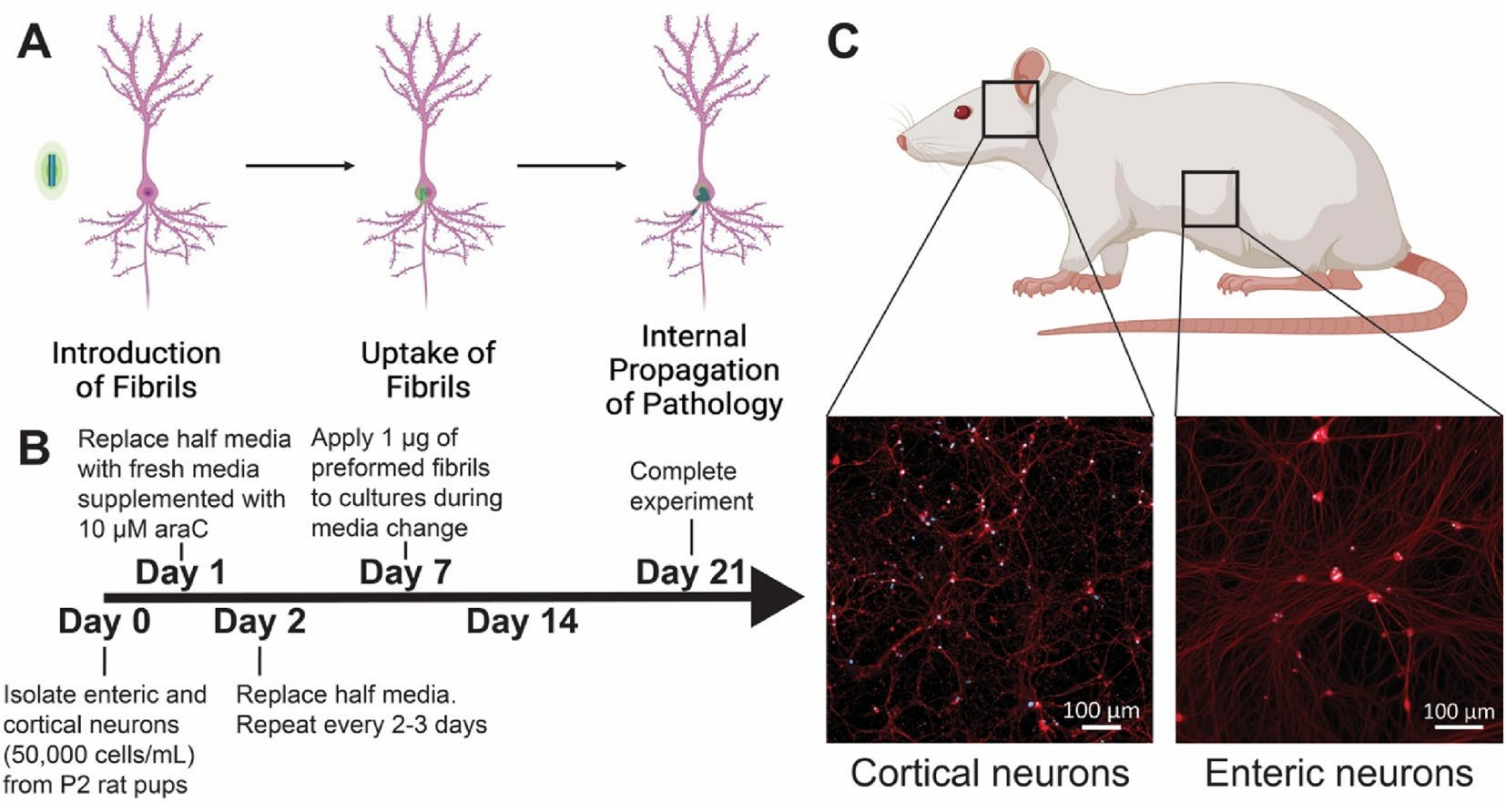
Schematic representation of the experimental design. (**A**) Illustration of the theorized preformed fibrils (PFF) toxic progression from the introduction of PFFs to uptake into neurons and development of further aggregation from endogenous α-synuclein (a-Syn). (**B**) Timeline of experimental design. Cultures were fed every 2–3 days throughout the experimental timeline. (**C**) Representative images of neuron populations (cortical and enteric) isolated for experimental procedures illustrated linking to anatomical location. Images are merged (Red = beta-3 tubulin, Blue = DAPI). Scale = 100 µm. Figure created with BioRender.

Preformed fibrils (PFF) of α-synuclein (a-Syn) have previously been reported to induce Lewy pathology (LP) by providing seeds for aggregation of endogenous a-Syn (Fig. 1A)^1,3,13,19^. While several works have established PFF aggregation in cortical neurons, limited assessments have been made with enteric cultures (implicated in PD pathogenesis). Thus, a comparison of enteric to cortical pathology may confirm the sensitivity of aggregation detection. In this work, fluorescently tagged (ATTO-594) PFFs were used to allow evaluation of PFF uptake and retention over time noninvasively.

Parkinson’s disease (PD) motor symptoms are believed to present following initial degeneration of the largely dopaminergic substantia nigra and then pathologically progress throughout the brain^1^. At the most advanced stages, the pathology reaches the cortex. Due to the small size of the substantia nigra and difficulty in isolating, cortical neurons are often used as primary cell models for CNS PD^1^ and were utilized in this work. Enteric and cortical neurons were isolated from P2 rat pups and cultured for 7 days prior to administration of 1 µg of human PFFs (Fig. 1B). On day 1, 10 µM of cytosine β-d-arabinofuranoside was dosed to reduce glial contamination. Following 21 days, neurons developed substantial neural outgrowth demonstrated by beta-3 tubulin immunostaining (Fig. 1C). Parkinson’s disease formation is linked to aging and most likely a result of chronic toxin exposure (e.g., pesticides). Our findings are from neonatal tissue and a single PFF exposure dosage^1,13,27,28^. Inclusion of experimental groups with butyrate and lipopolysaccharide (LPS) (detailed in sections "Live-imaging assessment of PFF delivery to neural cultures" and "Immunostaining analysis for PFF retention and aggregated a-Syn") aim to recapitulate aspects of a chronic inflammation in the gut.

### Live-imaging assessment of PFF delivery to neural cultures

**Figure 2:**
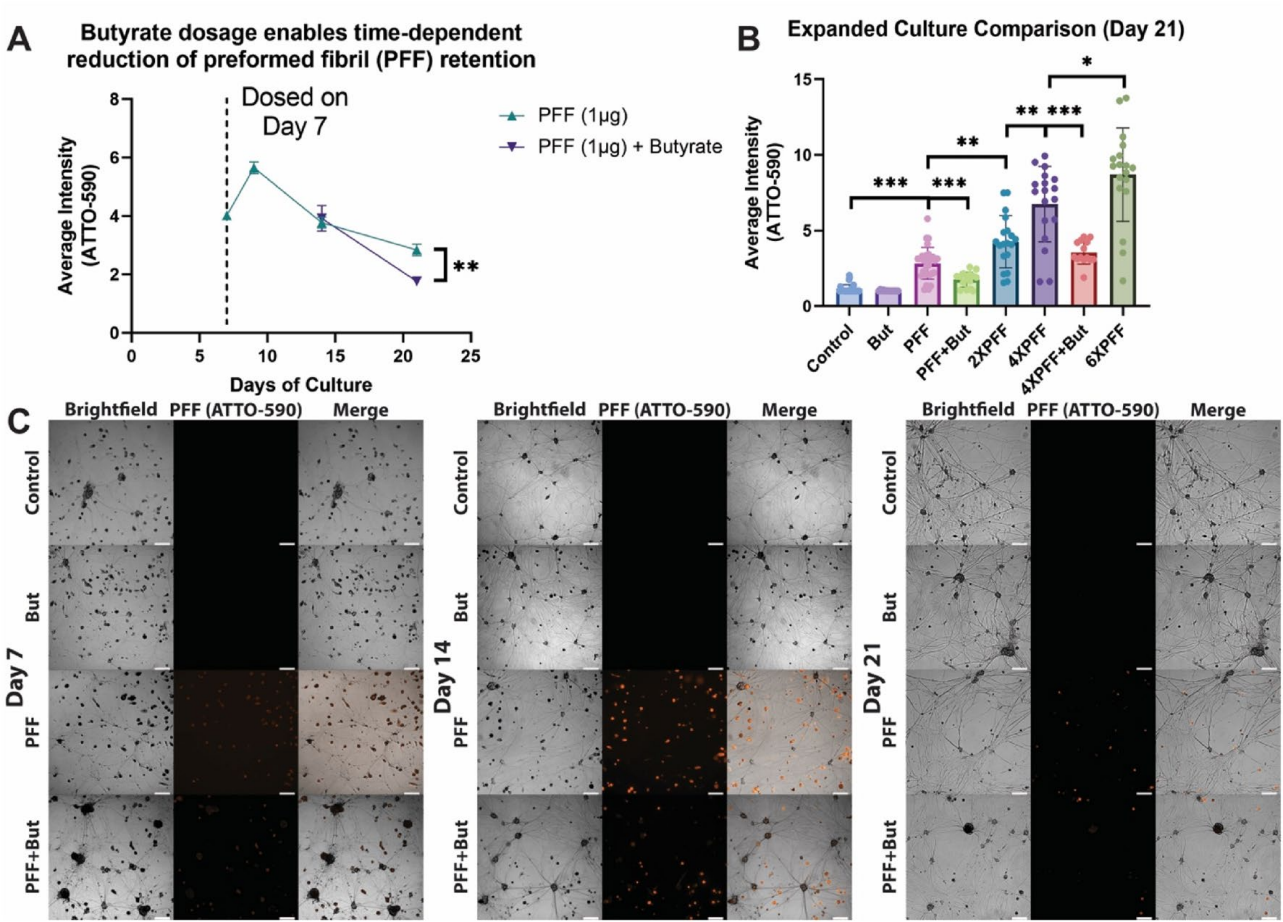
Live-cell imaging quantification of preformed fibril (PFF) intensity throughout experiments. (**A**) Average intensity of images across culture period. Box-cox transformed multilevel model accounting for experimental replicate and well with Tukey p-value adjustment used. N = 3–6, m = 9–21, images = 15–31. (**B**) Average intensity of images on day 21 of culture. Kruskal–Wallis test and Wilcoxon rank-sum multiple comparisons with Benjamini–Hochberg p-value adjustment utilized. N = 3–6, m = 8–20, images = 13–29. (**C**) Representative images of cultures over time between groups (orange = PFF fluorescent tag). Day 7 background signal is attributed to free-floating PFF after addition. (*p < 0.05, **p < 0.01, ***p < 0.001). Scale = 100 µm. Pixel intensity in uint8 (values range from 0 to 255). Each dot represents an image, error bars = SEM.

On days 7, 9, 14, and 21, live-cell fluorescent microscopy was used to assess the presence of PFFs within enteric neuron cultures. As lower levels of butyrate-producing bacteria have been linked to increased PD risk^1,24,25^, we aimed to model the relative presence of these bacteria by dosing 2.5 mM sodium butyrate (But) with every media exchange beginning upon PFF administration (day 7). Values were assessed with a box-cox transformed multilevel model adjusting for experimental replicate and well with Tukey p-value adjustment. As expected, the average intensity of the fluorescent PFF tag significantly rose following 1 µg initial dosage before declining throughout the remaining experiment (Fig. 2A, Supplementary Fig. S2). Notably, the 1 µg PFF with butyrate (PFF + But) group did not significantly differ from the 1 µg PFF group on day 14 (0.045 uint8 decrease, *p* = 1.0), only day 21 (0.532 uint8 decrease, *p* < 0.0049).

Next, we assessed an expanded number of groups on day 21 with live imaging using a non-parametric Kruskal–Wallis test and pairwise Wilcoxon rank-sum tests for multiple comparisons with Benjamini–Hochberg p-value adjustment (Fig. 2B). All PFF-dosed groups had significantly increased average PFF tag pixel intensity than controls (*p* < 0.0001 for all conditions). Butyrate dosage alone was not significantly different in average PFF tag intensity than the untreated control condition. Average PFF tag intensity was significantly altered between subsequent dosages (1 µg PFF–2 µg PFF, *p* = 0.0016; 2 µg PFF–4 µg PFF, *p* = 0.0014; 4 µg PFF–6 µg PFF, *p* = 0.0159), with an overall dose-dependent relationship (adjusted R^2^ = 0.6839, *p* < 0.0001, Fig. 2B, Supplementary Fig. S1C). In line with previous findings, 4 µg PFF with butyrate average PFF tag intensity was significantly lower than 4 µg PFF alone (*p* = 0.0004).

One explanation of why PFF average intensity rose from day 7 to day 9 could be that PFFs were primarily dispersed in the media but not yet uptaken by neurons on day 7. Thus, once they were imaged again 48 h later (day 9), the PFFs had largely transported into neurons, with the remaining PFFs being removed with each feeding or broken down by cells. This hypothesis is supported by a qualitative assessment of representative brightfield-fluorescent images (Fig. 2C, Supplementary Fig. S2) demonstrating a diffuse yet elevated fluorescent signal on day 7. An assessment of plotted maximum intensity pixels for each image over the culture period of the PFF group further supports this hypothesis (Supplementary Fig. S1).

These PFF uptake and retention observations over an extended culture period align with previous reports. Braidy et al. reported in primary human fetal enteric neurons that significant uptake of PFFs occurred at 30 and 60 min following PFF administration (measured via optical density)^15^. Additionally, they found uptake was impacted by endocytosis, proteasomal, and lysosomal inhibitors. While not tracking on the scale of hours following PFF dosage, we did observe PFF uptake within the first 48 h. Abounit et al. demonstrated fluorescently-tagged PFFs entered neuron-like cancer cells (SH-SY5Y) and localized in lysosomes, where they aggregated into a-Syn inclusions and transferred to neighboring cells^29^. Using direct stochastic optical reconstruction microscopy, Apetri et al. also reported PFF uptake and localization to lysosomes in SH-SY5Y cells^16^. However, they did not observe aggregation in lysosomes or the cytoplasm. Instead, they reported a slight shortening of fibrils. They used a lower concentration (0.1 µM) than this work (1 µM) or Abounit et al. (0.5 and 1.0 µM)^29^, which suggests cells can handle a set level of aggregation without pathological degeneration. These works support our findings that enteric neurons uptake and retain PFFs and that retention can be modulated.

A possible reason why butyrate co-administration reduces PFF retention on day 21, not 14, could be that butyrate assists in PFF processing rather than restricting initial uptake. In vivo PFF-based studies have reported reversal of a-Syn aggregation following initial formation^3,30,31^. For example, Manfredsson et al. reported initial PFF transmission to the brainstem one month following PFF injection to the myenteric layer of rats (colon) and non-human primates (colon and stomach) that was not observed in later time points, supporting neural capability to clear aggregated alpha-synuclein^32^. The reduction of PFF uptake into lysosomes as well as the reduction of lysosome function (i.e., cysteine protease inhibition via chloroquine) improves cell viability^33,34^. Butyrate has also been used to differentiate cancer cell-line cultures to a “dopaminergic” phenotype (1 mM dosage) via inhibition of histone deacetylase (HDAC)^35^. Histone acetylation is an epigenetic modification linked with aging and neurodegenerative disease^36–38^. Toker et al. reported elevated histone acetylation in idiopathic PD patients compared to healthy control patients^36^. Paiva et al. showed that butyrate rescued a-Syn induced transcriptional dysregulation and improved DNA damage in an in vitro A30P genetic PD model^38^. As increased butyrate levels have been linked to positive effects to PD^1,24–26^, future work must parse out how butyrate may support a reduction of PFF tag signal and whether such clearance induces or avoids subsequent toxicity. For example, quantifying the transcriptional and epigenetic impacts of butyrate on PFF-dosed cultures alongside oxidative damage (lipid peroxidation assay) and cytokine assays could be one route.

### Immunostaining analysis for PFF retention and aggregated a-Syn

**Figure 3:**
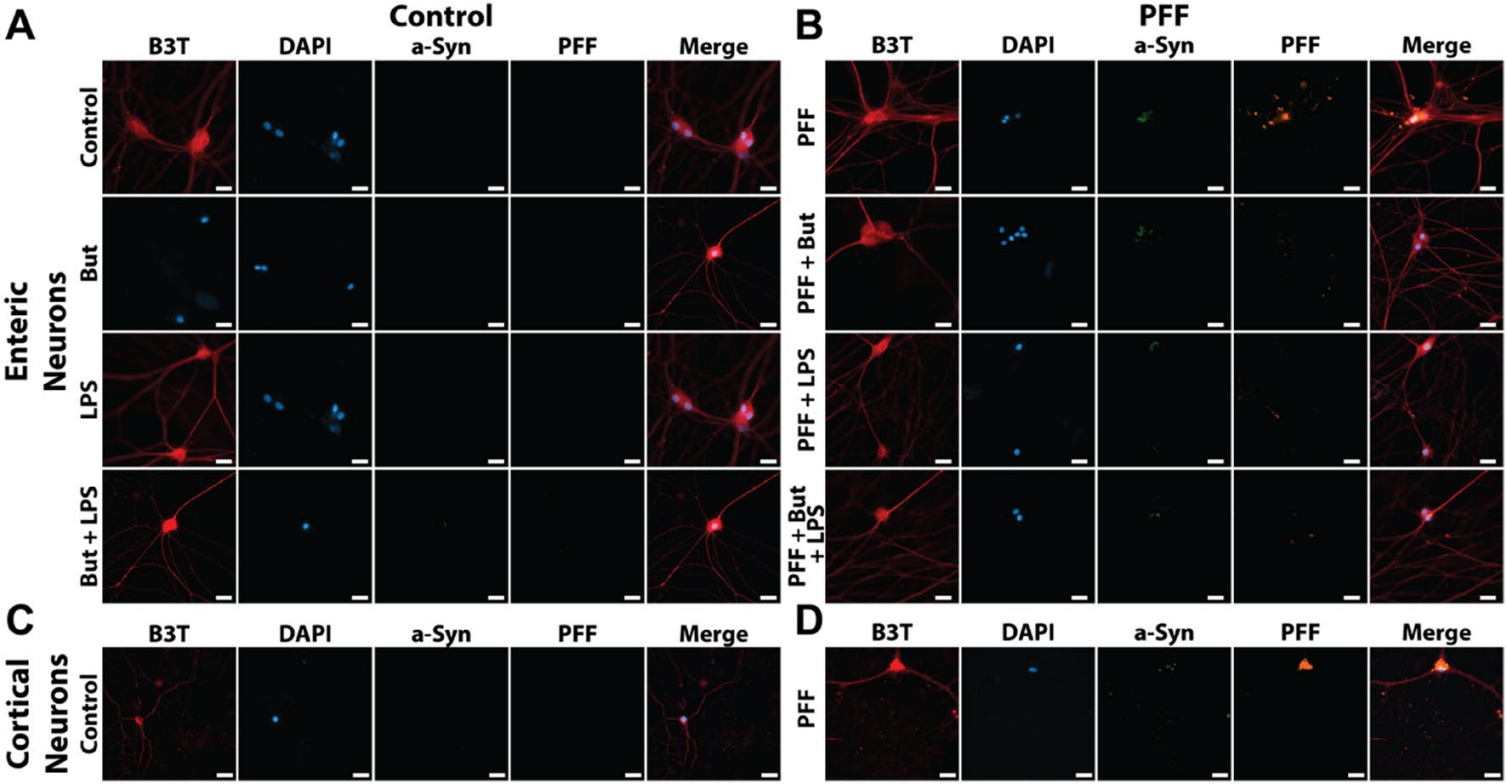
Representative images of neuron populations following 21 days of culture. ENS cultures were co-administered with LPS and/or butyrate either (**A**) alone (control), or (**B**) with PFFs. (**C**) Untreated cortical neurons. (**D**) PFF-dosed cortical neurons. (red = beta 3-tubulin (B3T), blue = DAPI, green = aggregated α-synuclein (a-Syn), orange = preformed fibril fluorescent tag). Scale = 20 µm.

We utilized immunostaining following 21 days of culture (14 with PFFs) to determine whether elevated aggregated a-Syn signal—indicative of pathological a-Syn inclusions found in LP—was observed. As expected, representative untreated (control) enteric and cortical neuron images show a characteristic beta-3-tubulin (B3T) morphology absent PFF fluorescent tag signal, with increased aggregated PFF fluorescent tag signal present in PFF treated cultures (Fig. 3, Supplementary Fig. S3). Aggregated a-Syn is present in representative cortical cultures (Fig. 3D) and co-localized with PFF fluorescent tag in the PFF group. Cortical culture aggregated a-Syn signal contained a morphology composed of spotted a-Syn positive inclusions 1/6th to 1/8th nucleus diameter, in line with a recently published protocol^39^. Enteric neuron cultures also retained elevated PFF fluorescent tag signal in PFF-dosed cultures. However, enteric neuron cultures displayed high variance in aggregated a-Syn signal with underlying overall trends between groups. In general, PFF-only conditions (1–6 µg) along with LPS-only cultures had elevated aggregated a-Syn signal compared to untreated cultures demonstrated by representative images (Fig. 3A,B). LPS cultures with co-administered butyrate did not have elevated levels of aggregated a-Syn.

**Figure 4:**
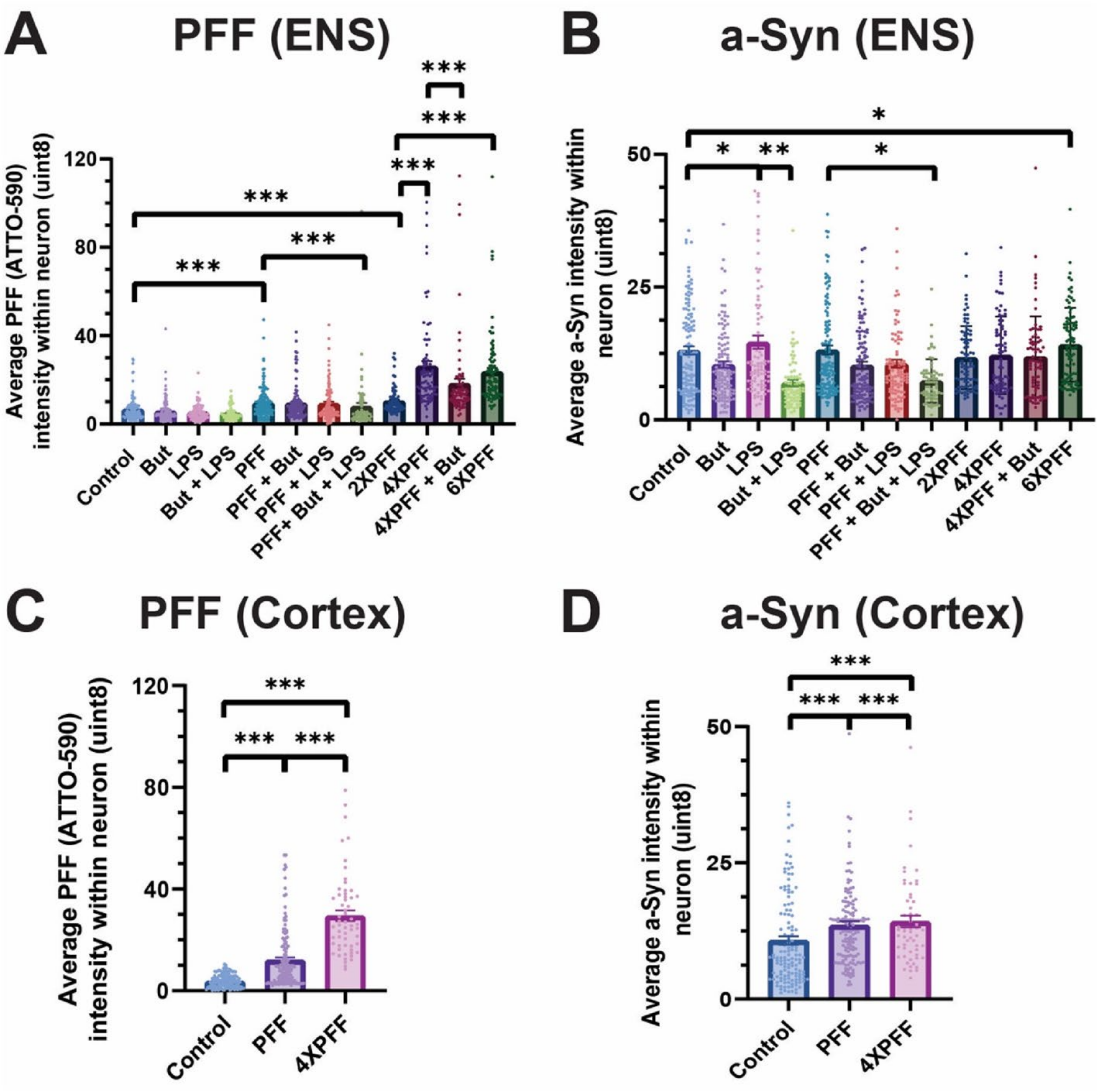
Quantification of preformed fibril (PFF) tag and aggregated α-synuclein (a-Syn) intensity throughout experiments. 2.5 mM of Butyrate (But) and 5 µg/mL lipopolysaccharide (LPS) were added with every feeding following PFF administration. The average intensity of (**A**) PFF and (**B**) aggregated a-Syn within ENS neuron morphology. Sample size ENS: N = 3–7, m = 9–18, images = 60–123. The average intensity of (**C**) PFF and (**D**) aggregated a-Syn within cortical neuron morphology. Sample size cortex: N = 4–8, m = 12–27, images = 57–131. Pixel intensity in uint8 (values range from 0 to 255). Each dot represents an image, and error bars = SEM. Statistical analysis was conducted using a 6th root transformation multilevel model adjusting for experimental replicate and well (**A**) or experimental replicate alone (**B**–**D**) with Tukey p-value adjustment (*p < 0.05, **p < 0.01, ***p < 0.001).

The immunostained pixel intensity was quantified within a neuron morphology mask identified from B3T and DAPI signal via MATLAB. For statistical assessment of ENS and cortical cultures, we used a 2- or 3-level multilevel model adjusting for experimental replicate and well or experimental replicate alone based on the likelihood ratio test. Enteric neurons had a significantly increased average pixel intensity for PFF tag signal (Fig. 4B) between control and each PFF-only group dosage (1 µg: 3.29 uint8 increase, *p* < 0.0001, 2 µg: 3.70 uint8 increase, *p* < 0.0001, 4 µg: 12.57 uint8 increase, *p* < 0.0001, 6 µg: 14.42 uint8 increase, *p* < 0.0001). The difference between 1 µg and 2 µg PFF was not significant (0.41 uint8 increase, *p* = 1.0), 2 and 4 µg PFF was quite significant (8.87 uint8 increase, p < 0.0001), and 4 and 6 µg PFF was not significant (1.27 uint8 increase, *p* = 0.89). Next, comparing aggregated a-Syn average intensity signal (Fig. 4A), the addition of LPS compared to controls resulted in a significant increase in average intensity (2.80 uint8 increase, *p* = 0.0286), and butyrate and LPS administered together decreased the average intensity compared to LPS alone (3.40 uint8 decrease, *p* = 0.0068). 6 µg PFF dosage increased average aggregated a-Syn intensity compared to untreated controls (2.4 uint8 increase, *p* = 0.0420).

Cortical neurons were analyzed in the same manner as enteric neurons. The average intensity of the PFF tag within neuron morphology (Fig. 4D) was significantly different between all groups (*p* < 0.0001). Aggregated a-Syn average intensity within neuron morphology of cortical neurons (Fig. 4C) was significantly increased between control and PFF groups (control—PFF (1 µg): 1.88 uint8 increase, *p* = 0.0027, control—PFF (4 µg): 9.01 uint8 increase, *p* < 0.0001). The difference between the 1 and 4 µg PFF dosage was also significant (7.13 uint8 increase, *p* < 0.0001). Estimated means plots of the average intensity analyses demonstrate relationships between groups (Supplementary Fig. S4).

Image analysis for each morphological structure subtype (whole image, neurite, soma, neuron) was conducted to parse out whether changes in intensity were driven by specific regions (Supplementary Fig. S5). The whole image (i.e., no morphological mask) analysis resulted in a higher number of statistically significant aggregated a-Syn average intensity differences between experimental groups, with no difference in the number of statistically significant PFF average intensity relationships. In the neurite morphology, the significant relationships in average PFF tag intensity were the same as the neuron morphology and whole image analysis, except that the difference between 1 µg PFF and 1 µg PFF with butyrate and LPS was not significant. Contrarily, in the soma region, one more significant relationship in PFF tag average intensity was observed (1 µg PFF–1 µg PFF + LPS, 4.4 uint8 decrease *p* = 0.0265). Notably, while whole image analysis of cultures identified a significant increase in average aggregated a-Syn intensity between untreated and 1, 2, and 6 µg PFF groups average intensity (1 µg PFF: 2.00 uint8 increase, *p* < 0.0001; 2 µg PFF: 1.27 uint8 increase, *p* = 0.0007; 4 µg PFF: 0.64 uint8 increase; *p* = 0.47; 6 µg PFF: 2.21 uint8 increase, *p* < 0.0001), a more limited number of significant relationships were found in the neurite, soma, or neuron morphology analyses. In addition, the whole image analysis also detected an increase in aggregated a-Syn average intensity in LPS cultures compared to untreated controls (1.77 uint8 increase, *p* < 0.0001) that was ameliorated by co-administration of butyrate (1.46 uint8 decrease, *p* = 0.0100), which was also detected in the neurite and neuron morphologies, but not the soma. In the neurite region analysis, there was a significant increase in aggregated a-Syn average intensity in the 6 µg PFF group compared to untreated controls (2.21 uint8 increase, *p* = 0.0177). The soma morphology exhibited a significant decrease in aggregated a-Syn average intensity between 1 µg PFF and 1 µg PFF groups with butyrate and LPS (7.3 uint8 decrease, *p* = 0.0002).

Additionally, we complementarily evaluated the area of elevated signal within images for both PFF tag and aggregated a-Syn channels for all morphologies in ENS (Supplementary Fig. S6) and within neuron morphology of cortical cultures (Supplementary Fig. S4E,F). Cortical cultures demonstrated nearly identical relationships (except the 1 µg PFF and control relationship was not significant). 4 µg PFF dosage in ENS cultures resulted in a significant increase in the fraction of aggregated a-Syn elevated area (*p* = 0.0490) compared to untreated controls. However, no significant relationship between LPS and the control group occurred. PFF tag elevated area within the ENS neuron morphology demonstrated a PFF dosage-dependent relationship, which butyrate reducing PFF tag elevated area at high dosage (4 µg PFF + But–4 µg PFF: *p* < 0.0001). Further information on relationships is available in supplementary materials.

We investigated whether nuclei count (via CellProfiler, Supplementary Fig. S7) impacted aggregated a-Syn signal. Plotted comparisons of nuclei count against average intensities of aggregated a-Syn and PFF tag for untreated ENS neurons (Supplementary Fig. S8A–D) lacked a clear relationship between each metric. For example, the highest PFF or aggregated a-Syn average intensity values occured in images with up to 30 counted nuclei, where the range of counted nuclei was 0–115. Adjustments of statistical analyses for nuclei count did substantially improve the detection of significant relationships (Supplementary Fig. S9).

Our findings suggest that PFFs are retained in both ENS and cortical cultures, in line with live imaging quantification. Cortical cultures demonstrated a significant increase in average PFF tag and aggregated a-Syn intensity in groups dosed with PFFs and between PFF doses (1 and 4 µg). Contrary to the cortical cultures, ENS cultures were less responsive within the neuron morphological region. While whole image analysis of cultures identified a significant increase between untreated and 1, 2, and 6 µg PFF groups in aggregated a-Syn average intensity, only the highest dosage (6 µg) was significant within the neuron morphology. These findings suggest aggregation of a-Syn occurred in the cortical neuron cultures, as reported in previous works^17,19^, and within the highest PFF dosages of ENS cultures. Conformation-specific a-Syn aggregation antibodies (including ab209538 utilized in this work) may possess insufficient specificity, which could represent a limitation of our study design^40^. Further experimental analysis with phosphorylated a-Syn and thioflavin staining, and proteinase K digestion would strengthen our observations and allow for more inference into PFF processing related to butyrate.

Our results show that LPS dosage increases aggregated a-Syn average intensity, suggesting LPS may induce a-Syn aggregation. This observation is in line with work that indicates that bacterial translocation via LPS-detecting TLR4 activation can induce α-synuclein in rodent models^41^. The effect of LPS addition is ameliorated by butyrate, which supports the modulatory impact observed in live imaging with PFF dosage. Although butyrate appears to result in an insignificant decrease in aggregated a-Syn average intensity with PFF dosage, it may be that more time is required for a significant decline to be detected, as a significant decrease in live average PFF tag intensity did not occur until day 21. LPS dosage, when co-administered with PFFs, resulted in a decrease in aggregated a-Syn in the soma morphology, in line with published comparisons of in vivo model findings, where the presence of endotoxin in PFFs can result in a decrease in uptake^1,19^.

Limited work on PFF-induced a-Syn aggregation in enteric neurons in vitro is available to date. Lassozé et al. recently analyzed the detection of ENS a-Syn aggregation via western blot with five phosphorylated a-Syn antibodies^21^. Braidy et al. observed PFF uptake in primary human fetal enteric neurons and the impact on mitochondrial activity but did not explore a-Syn aggregation^15^. Gries et al. reported a significant decline in viability and neural (via protein gene product 9.5) cells per image in mice enteric neurons 5 days following mutant PFF dosage^22^. They did not investigate a-Syn aggregation. While they used a lower concentration than this work (0.5 µM compared to 1 µM), their PFFs were A390P mutants (a genetic form of PD), which may be more efficient at inducing pathology than the wild-type protein. In non-PFF models, Pan-Montojo et al. reported that non-neuronal cells neighboring primary enteric neurons had significantly more a-Syn inclusions following rotenone dosage but did not report quantified results on a-Syn aggregation^13^. In primary hippocampal neurons, Shrivastava et al. reported differences in a-Syn aggregation (measured as area-covered and total fluorescence) depending on fibril polymorphs^42^. They observed a similar effect size shift in fluorescent intensity 14 days following PFF dosage, however with a much lower percent area covered by a-Syn, although they used a phosphorylated a-Syn stain, not an a-Syn antibody^1,42^. Additionally, they reported different effect size responses between 1.5 µM dosages of fibrils, ribbons, and fibrils-91, modulated by PFF dosage, for both total fluorescence and percent area covered, indicating the unique differences which may be present with different fibrils. The literature to date mainly predicts that the ENS can form a-Syn aggregation following PFF dosage or environmental stimulus, in line with our observations in the 6 µg PFF and LPS experimental conditions. While different primary neuron cultures from different brain regions uptake similar levels of exogenous a-Syn, endogenous expression has been shown to impact the vulnerability of populations to exhibit LPs^43^. Future work should include a direct comparison of endogenous a-Syn levels in both ENS and brain primary cultures.

### Assessment of growth cone morphology and dynamics

**Figure 5:**
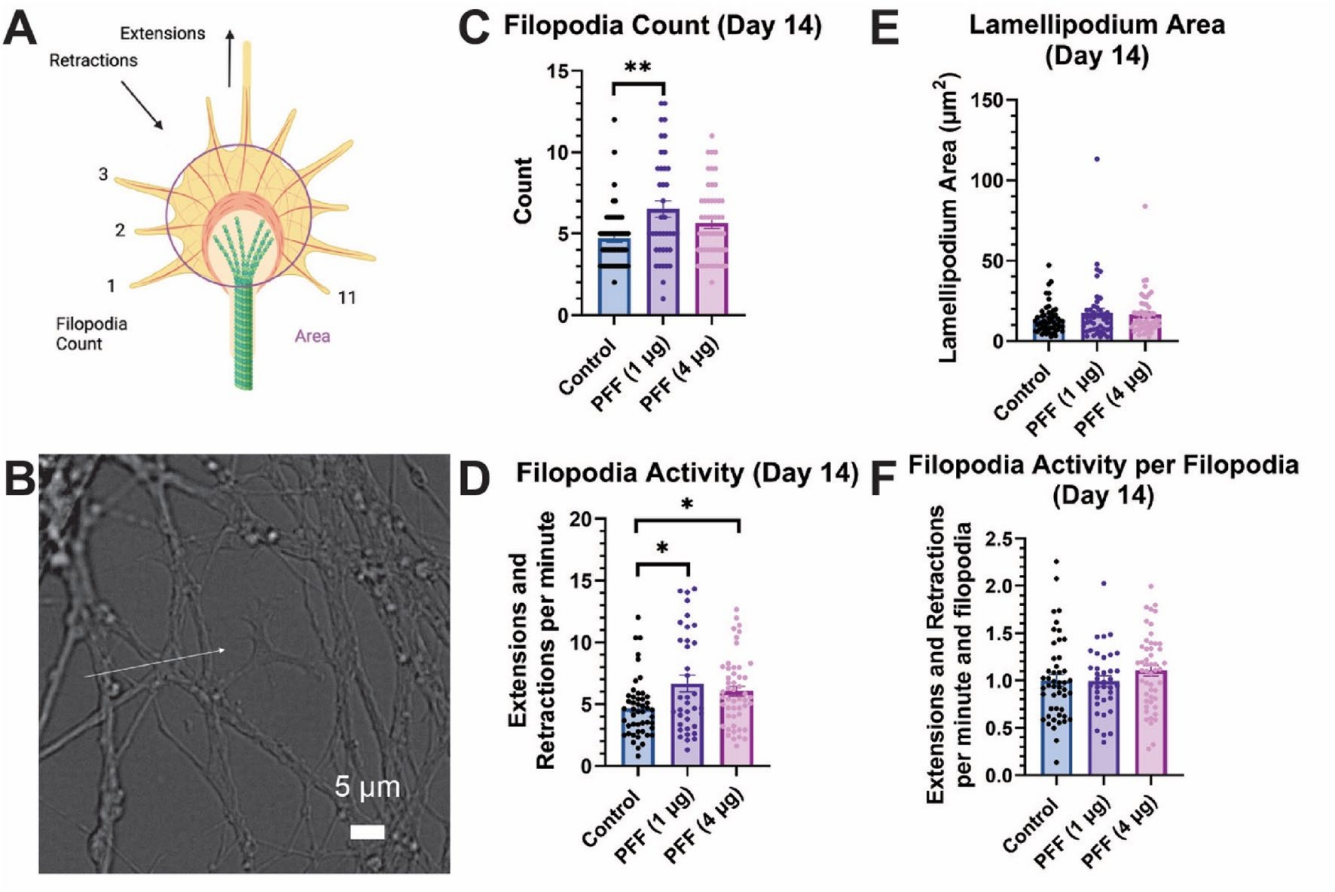
Growth cone quantitative assessment of ENS cultures observed on day 14 of culture. (**A**) Growth cone representative diagram. Figure made with Biorender. (**B**) Representative brightfield image of a growth cone. Day 14 measurement of (**C**) filopodia count, (**D**) filopodial activity (sum of extensions and retraction events per time passed), (**E**) lamellipodium area, (**F**) filopodial activity normalized per filopodia. Statistical analyses were conducted with either logarithmic (**C**,**E**) or 4th root (**D**,**F**) transformation and either general linear model (**D**,**E**) or multilevel model adjusting for experimental replicate and well (**F**) or replicate alone (**C**). N = 4, m = 11–12, growth cones = 43–54. Each dot represents a growth cone. Error bars = SEM. (*p < 0.05, **p < 0.01, ***p < 0.001).

Increasing evidence supports decreases in microtubule expression in neurons associated with PD or PD disease models^22,44^. Growth cones—which form and elongate neurites and axons—serve as a physiologically important site where reported microtubule expression may be translated to functional impacts associated with the disease^44,45^. a-Syn has been shown to regulate microtubule dimerization in growth cones and is localized to the region^45^. Postmortem analysis of PD patient brains indicated a decrease in dendritic spine density in medium-sized spiny neurons and caudate nucleus dendritic tree size^46^, supporting the investigation of whether growth cone dynamics are altered following PFF dosage. Although, to date, several works have linked growth cone dynamic changes to neurodegeneration, limited work has focused on PD-related modulation of growth cone functionality, particularly for PFF dosage^47–49^. Here, we aimed to understand whether enteric neurons dosed with either 1 µg or 4 µg PFF had changes in filopodia count, filopodial extension/retraction rate (both raw and normalized to filopodial count), or lamellipodium area (Fig. 5, Supplementary Fig. S10).

We assessed differences between cultures cross-sectionally using either a log (filopodial count and lamellipodium area) or 4th root (filopodia activity and normalized filopodia activity) transformed general linear model or multilevel model depending on the likelihood ratio test. We observed significantly more filopodia per growth cone (Fig. 5C, Supplementary Fig. S10) in 1 µg PFF cultures compared to untreated control populations on day 14 (1.45 more filopodia, *p* = 0.0054) and 21 (1.45 more filopodia, *p* = 0.0057). There was no significant difference between untreated control populations and the 4 µg PFF population (day 14: 0.61 more filopodia, *p* = 0.1611; day 21: 0.77 more filopodia, *p* = 0.0878). No statistically significant differences in lamellipodium area between groups were detected (Fig. 5D). We observed a significant increase in filopodial extensions and retractions per minute (Fig. 5E) for both PFF dosages on day 14 (1 µg PFF: 1.52 increase, *p* = 0.0163; 4 µg PFF: 1.23 increase, *p* = 0.0304). Normalizing the filopodial extension/retraction rate for each growth cone by dividing by the number of filopodia, we observed no statistically significant differences between experimental groups (Fig. 5F).

Our results suggest that PFF dosage may result in morphological changes in growth cones reflected by increased filopodia count. Previous growth cone literature related to PD has been limited. Schechter et al. reported that A53T a-Syn mice had a higher axonal density (number of axons per µm^2^ and lower axonal diameter but the same growth cones per axon frequency^47^. Since a-Syn functions to regulate growth cone microtubule dimerization^45^, a possible reason for the observed changes in growth cone morphology could be that PFF dosage impacts underlying growth cone mechanics towards multiple smaller neurites reflected by a higher number of filopodia. These findings support our observations of increased filopodial count per growth cone. Future work, including longer incubation time and fluorescent membrane reporting (to allow for more complex quantification), could elucidate whether changes occur in growth cone dynamics.

### Electrophysiological recordings via microelectrode array

Existing literature has reported mixed levels of electrophysiological decline following PFF dosage (often in cortical neurons). No work has yet described to our knowledge whether enteric neurons respond similarly, although clinical meta-analysis indicates constipation may precede PD symptoms by decades^7–9^, implicating ENS electrophysiological degeneration. Physiologically, the enteric nervous system (ENS) is a heterogenous population with at least 14 types of neurons and numerous neurotransmitters, including dopaminergic and cholinergic neurons, which conduct a wide range of functions, including gut motility^50–53^. Peristalsis is maintained through a complex excitation and inhibition pattern, dominated by cholinergic (acetylcholine) and nitrergic (nitric oxide) neurons, respectively^52,53^. Recent work has suggested dopaminergic neurons play a role in peristalsis through regulation of nitrergic activity^53^.

**Figure 6:**
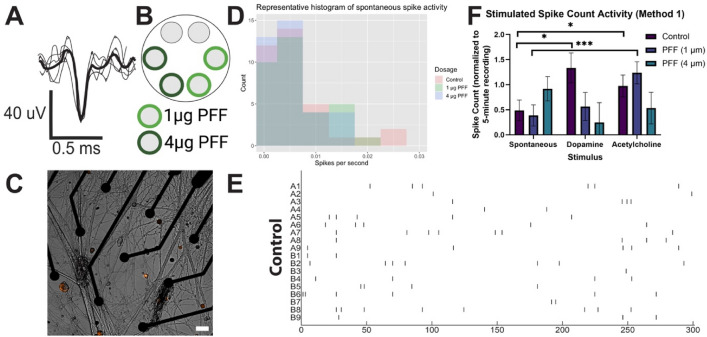
Functional assessment of neurons using microelectrode arrays (MEA). (**A**) Representative waveform (**B**) Schematic made in Biorender. (**C**) Merged image of neurons near 30 µm diameter electrodes (black). Orange = PFF tag. Scale = 100 µm. (**D**) Representative histogram of detected action potential rate as spikes per second from spontaneous activity recorded on day 20 (upper 7% of values cropped). Color indicates experimental group (red = control, green = 1 µg PFF, blue = 4 µg PFF). (**E**) Representative raster plot of spontaneous spike activity over time (seconds). (**F**) Estimated means of spike count (5-min period) by stimulation and PFF dosage (method 1). Error bar = SEM. N = 1–4, number of MEAs = 1–4, wells = 2–11, number of electrodes = 10–50. (N > 1 outside day 28, 4 µg PFF. Zero-inflated, negative binomial generalized multilevel model adjusting for culture day and nested random effects for experimental replicate and electrode with Tukey p-adjustment was used. (*p < 0.05, **p < 0.01, ***p < 0.001).

Since PD is well-known for targeting dopaminergic pathways^1^, yet the ENS is a diverse population, understanding whether stimulated activity differs by PFF dosage could offer insight into PD pathogenesis. For this reason, we trialed several stimuli using two methods to ensure electrophysiological differences were holistically compared (Supplementary Fig. S12). Acetylcholine was chosen for prevalence and role in gut motility^50,52^. Dopamine was selected for its pathological link to PD^50,51,53^. Second, we aimed to model the pacemaking activity of local interstitial cells of Cajal (ICCs) using electrical stimulation with a square waveform (1200 mV for 1000 ms with 2000 ms break). The waveform was motivated by literature using dorsal root ganglia^54^ and ex vivo microelectrode recordings of rodent interstitial cells of Cajal^55^. We theorized that PFF dosage would result in decreased electrically-induced action potential activity, defined as a spike. Thus, on day 20, dopamine was added after electrical stimulation to test the cumulative role of electrical and dopamine stimulus by PFF dosage. Neurons were seeded onto 6-well microelectrode arrays (MEAs) to assess electrophysiology with PFFs (1 and 4 µg) introduced to cultures on day 7 (Fig. 6A) and confirmed via live imaging (Fig. 6B,C, Supplementary Fig. S11).

Measured spike activity was highly heterogenous (Fig. 6D, Supplementary Fig. S13), with only 0.3% of recorded electrodes displaying bursting activity, defined as 4 spikes in 0.1 s (40 Hz)^56^. We used a zero-inflated, negative binomial generalized linear multilevel model adjusting for culture day and nested effects of experimental replicate and electrode with Tukey p-value adjustment to identify interaction effects of PFF dosage and stimulus condition on the number of spikes in a 5-min recording (Supplementary Fig. S14A).

Analyzing results from the neurotransmitter-only stimulus experiment (method 1, Supplementary Fig. S12 (method 1)), we identified no change in spontaneous spike count in either 1 or 4 µg PFF groups compared to untreated controls (Fig. 6F). We observed that stimulated spike count in untreated controls was significantly increased compared to spontaneous activity via acetylcholine (0.486 spike increase, *p* = 0.0151) and dopamine (0.85 spike increase, *p* = 0.0144). In the 1 µg PFF dosage, acetylcholine stimulation significantly increased spike count compared to spontaneous activity (0.85 spike increase, *p* < 0.001), but not dopamine stimulus (0.15 spike increase, *p* = 1.0). In the 4 µg PFF dosage, no statistically significant effect of stimulation on spike count was observed compared to spontaneous activity for either acetylcholine (0.39 spike decrease, *p* = 0.91) or dopamine (0.67 spike decrease, *p* = 0.68). We observed that our pattern electrical stimulus (method 2, Supplementary Fig. S12) did not elicit a change in spike count between groups or stimuli (Supplementary Fig. S14C) using a negative binomial generalize multilevel model adjusting for nested effects of experimental replicate, MEA, and electrode with Tukey p-value adjustment.

These results suggest that in vitro cultured ENS experience limited alteration in spontaneous electrophysiological function and overcome PFF dosage to maintain consistent activity. This could partly explain how PD patients can host ENS LP up to several decades prior to motor symptoms without total loss of gut motility^1,9^. We observed PFF dosage-dependent relationships for stimulus–response. In control populations, dopamine stimulation increased spike count compared to spontaneous activity, while no statistical differences were observed in 1 and 4 µg PFF dosage cultures. Interestingly, following acetylcholine stimulation, spike counts in the control and 1 µg PFF dosage groups significantly increased, but 4 µg PFF cultures did not. These findings suggest that PFF dosage differentially impacts dopamine-responsive activity compared to acetylcholine-responsive activity. Since the ENS is more sparsely composed of dopaminergic neurons (~ 10% of enteric neurons^51^) compared to cholinergic neurons (~ 60% of enteric neurons)^57^ a decrease in spontaneous dopaminergic spike rate may be challenging to observe. Future work may better elucidate this relationship by either quantifying aggregation and survival by neural type or comparing single-cell transcriptional differences between cholinergic and dopaminergic enteric neurons.

To our understanding, these are the first in vitro MEA recordings in ENS cultures reported that investigated PFF impact. The available literature on the effects of PFF dosage via MEA recording among other cell populations has mostly reported decreases in spike activity. Peelaerts et al. observed an increase in inter-spike intervals of ex vivo cortical neurons^58^. They also observed a decrease in spontaneous burst frequency in primary cortical neurons exposed to PFFs via patch-clamp. Hassink et al. previously reported a decrease in mean firing rate in primary rat cortical neurons^59^, while Valderhaug et al.^60^ reported no significant change (both via MEA recording). Shrivastava et al. reported a significant drop in spike frequency among primary mouse hippocampal neurons via MEA recording^42^. We observe no difference in spontaneous spike count between PFF treated cultures and untreated controls but a significant difference in dopamine and acetylcholine stimulus–response.

### Phenotypic characterization of PFF dosed cultures

To further explore culture response to PFF dosage, we explored Substance P levels via ELISA quantification. Substance P functions with several purposes within the body, including neurotransmission in the ENS subtypes^50,61^. Increases in serum substance P have been closely linked to PD neurodegeneration. Shirinzi et al. reported that PD patients had an elevated level of substance P compared to healthy patients^61^.

**Figure 7:**
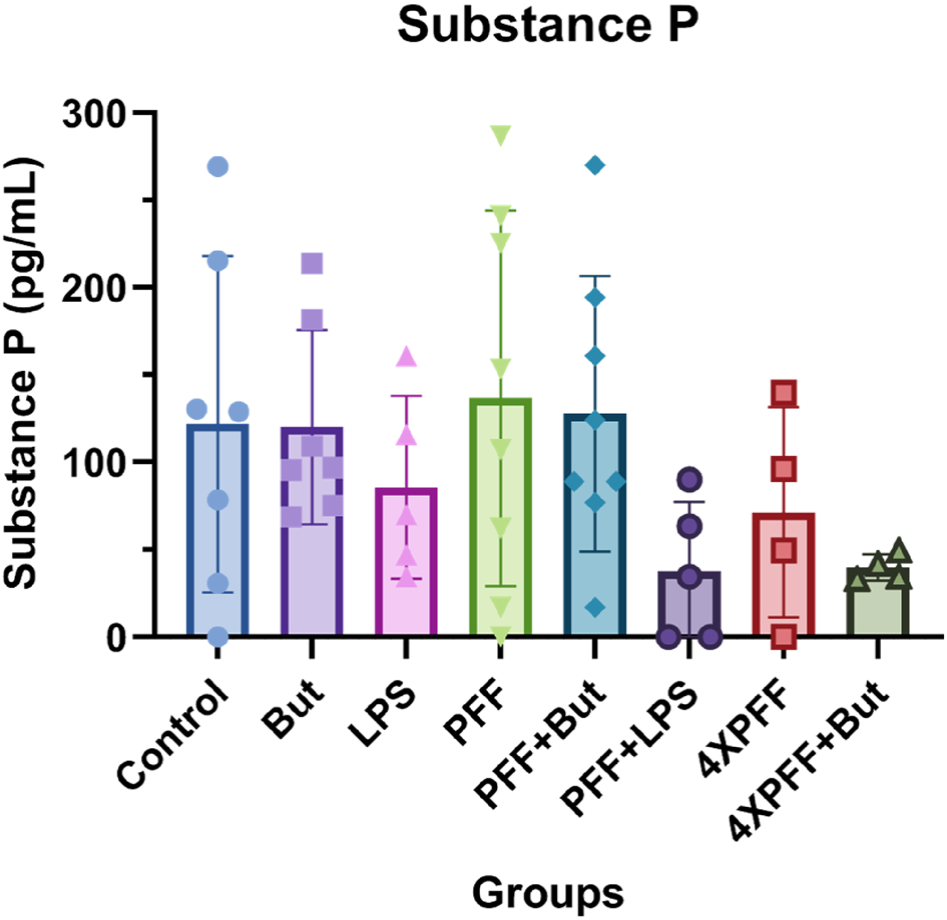
Substance P ELISA quantification of Day 21 spent medium demonstrates no significant differences between groups. N = 4–7. Error bars = SEM.

Our findings indicate that PFF dosage does not alter cell culture medium substance P levels (Fig. 7). These findings support our immunostaining and electrophysiological findings, which suggested no significant increase in aggregated a-Syn average intensity within neuron morphology below 6 µg PFF dosage and limited spontaneous impact of PFF dosage, respectively. These relationships could be further elucidated with a more extended culture time.

## Conclusion

Lewy pathology in the enteric nervous system (ENS) has been observed in Parkinson’s disease (PD) patients up to decades prior to motor symptom onset, presenting a need to understand how pathology develops and degenerates the ENS. Our findings suggest that the ENS and cortical neurons uptake preformed fibrils (PFFs) dose-dependently. Additionally, luminal contents such as butyrate may modulate ENS PFF retention. Fixed imaging analysis indicates that cortical neurons respond to multiple PFF dosages with an increase in aggregated α-synuclein (a-Syn), and ENS cultures only did at the highest dosage level (6 µg). ENS cultures dosed with PFFs had changes in growth cone morphology but not in growth cone dynamics. Electrophysiological recordings indicated no difference in spontaneous spike rate activity. However, a differential response of PFF-dosed neurons and untreated controls to dopamine stimulus compared to acetylcholine stimulus was observed. Finally, substance P media levels were unchanged following PFF dosage. These findings suggest that the ENS may largely avoid acute degeneration within our culture conditions. Future work should include aged, human, and chronically PFF dosed neural populations to provide insight on the enteric formation of and response to PD pathology.

## Experimental methods

### Neuronal cell culture

Isolated neurons were seeded on either 12 mm coverslips in 24 well plates, 24 well glass bottom plates, or 6-well microelectrode arrays (MEA) (cat: 890265, Harvard Apparatus) in a petri dish. Cell culture surfaces were prepared as follows. Coverslips were autoclave sterilized, and MEAs were UV sterilized for 10 min on top and bottom. Coverslips, wellplates, and MEAs were plasma-treated for 60–90 s. Culture surfaces were coated with 60 µL of 0.1 mg/mL poly-d-lysine (cat: A3890401, lot: 913158B Gibco), 60 µL of 0.2% w-v porcine gelatin (cat: G1890-100G, lot: SLBQ9498V, Sigma), and 60 µL of 40 µg/mL laminin (cat: 354232, Corning) each successively incubated at 37 °C for 1 h.

Enteric neuron (EN) media was composed of neurobasal-A (cat: 10888022, Gibco), 1× B27 supplement (cat: 17504044, Gibco), 1% fetal bovine serum (cat: MT35011CV, Corning), 1% antibiotic–antimycotic (cat: 15240062, Gibco), and 1% Glutamax supplement (cat: 35050061, Gibco). 0.1 ng/µL of glial cell line-derived neurotrophic factor (GDNF) (cat: PHC7045, Gibco) was added fresh prior to feeding or used within 7 days. For initial seeding and the first two feedings of the enteric neuron population cultures (Day 2 and 3), media was supplemented with 100 µg/mL of primocin (cat: ANTPM1, Invivogen) to eliminate any possible remaining contamination from isolation. Enteric neurons were fed on Day 1 with a half media exchange including 10 µM Cytosine β-d-arabinofuranoside (araC) (cat: C1768-1G, Sigma). Then on Day 2 with a half media exchange and every 2–3 days following. Cortical neuron (CN) media was composed of neurobasal-A (cat: 10888022, Gibco), 1X B27 supplement (cat: 17504044, Gibco), 1% penicillin–streptomycin (cat: 15140122, Gibco), and 1% Glutamax supplement (cat: 35050061, Gibco). 25 ng/mL of nerve growth factor (NGF) (cat: 13257-019, Gibco) was added to media fresh or used within 7 days. Cortical cultures were fed on Day 2 with a half media exchange including 10 µM araC. Then on Day 3 with a half media exchange and every 2–3 days following.

### Primary cell isolation

Primary enteric and cortical neurons were isolated from 2-day-old (P2) Sprague–Dawley neonatal rats (Charles River Laboratories). All experimental procedures involving animals were conducted in accordance with under protocols approved by Northeastern University's Institutional Animal Care and Use Committee and adhere to ARRIVE (Animal Research: Reporting of *In Vivo* Experiments) guidelines. Rodents were euthanized under approved protocol (19-0627R). Enteric neurons were isolated based on a previously detailed protocol from small intestines^62^. Cells were at 50,000 cells per coverslip or well and 17,000 or 50,000 cells per MEA well.

Isolated cortical tissue was digested at 37 °C for 30 min. Following incubation, the sample was diluted with 10.5 mL of DPBS and centrifuged at room temperature at 200G for 5 min. After removing the supernatant, tissue was resuspended in 5 mL of PBS and triturated 5× using progressively finer pores. The remaining cell suspension was put through a 40 µm cell strainer (cat: 22363547, Fisher) and centrifuged again at 200G for 5 min. The supernatant was removed, and the cell pellet was resuspended in 3 mL of CN media. Cells were seeded at 50,000 cells per well.

### Preformed fibril handling and application

Active, full length human recombinant preformed α-synuclein fibrils (PFFs) (cat: SPR-322C-A594, StressMarq Biosciences) tagged with the ATTO 594 were stored at – 80 °C based on previously available protocols^19,63^. PFF concentration was chosen as the recommended dosage by previous literature on cortical neurons^64^. On Day 7 of cell culture, PFFs were dosed into cultures during a media exchange at 2 µg/mL. Prior to dosage or handling PFFs were bath sonicated for 2 min at room temperature to ensure PFFs are seeded at a ~ 50 nm size. StressMarq datasheets report fluorescently conjugated vs. standard PFFs have similar seeding capacity via Thioflavin T seeding assay. Safety precautions were conducted in line with published recommendations^19,63,65^.

### Liposaccharide and sodium butyrate co-administration

On day 7, 1× (5 µg/mL) lipopolysaccharide (LPS) (cat: 00-4976-93, Invitrogen) and 2.5 mM sodium butyrate (But) (cat: 00-4976-93, eBioscience) were co-administered with PFFs within select experimental groups. Following PFF addition, LPS and But continued to be included in every media exchange until experimental completion.

### Immunostaining/imaging

Cells were fixed with 4% paraformaldehyde (cat no. 28906, ThermoFisher) for 20 min at room temperature, washed two times with DPBS, permeabilized with 0.1% triton-X (cat: X100-5ML, Sigma) for 10 min at room temperature, washed two times, and blocked for 1 h at room temperature with 2.5% goat serum (cat: G9023-10ML, Sigma). Next, antibodies (see Table 1) were applied in goat serum in two stages (primary and secondary) for 1 h each at room temperature, with 4 wash steps (at least 5 min each) following every application. Stained coverslips were mounted onto glass slides (cat: 22-037-246, Fisher) with ProLong™ gold antifade mountant with DAPI (cat: P36931, Invitrogen), incubated in darkness at room temperature for an hour, and stored at – 20 °C. Cultures were imaged using a Zeiss Axio observer Z1 inverted fluorescent microscope at 63× magnification.

**Table 1:** Immunostaining antibodies.

Protein target	Product number	Vendor	Wavelength (nm)	Host	Dilution
Beta-3 tubulin	Ab41489	Abcam	N/A	Chicken	1:1000
Aggregated a-Syn	Ab209538	Abcam	N/A	Rabbit	1:5000
Chicken	Ab150171	Abcam	647	Goat	1:1000
Rabbit	Ab150073	Abcam	488	Donkey	1:1000

### Immunostaining image analysis

Images were processed in biological replicate groups using MATLAB. Neural structure masks were developed for identifying neurites and somas to control for varying levels of neuron coverage, endogenous signal, and cell density differences in each image. Methodologically, binary masks were formed using morphological structuring elements from beta-3 tubulin and DAPI channel images. Average intensity was calculated for the whole image or within neurites, somas, or the combined neuron morphology masks.

### Live imaging of PFF retention and growth cone dynamics

All live imaging was conducted with immunostaining imaging using a Zeiss Axio observer Z1 inverted fluorescent microscope at 10X (PFF retention) and 63X magnification (growth cone dynamics) on an incubated stage (37C and 5% CO2). For live imaging of PFF retention, 4 groups were imaged at each timepoint to elucidate any modulatory impact of butyrate on cultures: control (no dosage added to media), butyrate (But), 1 µg preformed fibrils (PFF), and 1 µg PFF with butyrate (PFF + But). On day 21—prior to staining—an expanded number of experimental groups were imaged and compared, including a concentration gradient of PFFs from 1 to 6 µg and an elevated PFF dosage (4 µg) with butyrate (4XPFF + But). For growth cone dynamics recordings, images were taken about every second for 5 min on an incubated stage. If focal drift was too great, multiple shorter recordings were made to about 5 min of recording total. Analysis was conducted on ImageJ. Growth cone area and filipodia count were measured on the clearest image. The number of filopodia extension and retraction events was counted separately and added to form a single activity metric.

### Microelectrode array (MEA) recording and analysis

Cell cultures on 6-well MEAs (Multi-Channel Systems) were recorded in two methods detailed in Supplementary Fig. S12. Recordings typically totaled 4–6 min. A temperature-controlled plate was used to keep cell cultures at 37C. Electrodes were allowed to equilibrate for approximately 5 min before starting recordings. MC_Rack software from Multi Channel Systems collected digital voltage trace recordings from the MEAs.

Two methods were developed to elicit and identify changes in electrophysiological functioning to compare stimulated activity. The first method utilized 100 µM dopamine hydrochloride (cat: H8502-25G, Sigma) and 1 µM acetylcholine chloride (cat: A2661-25G, Sigma) with an hour washout incubation between stimuli to determine whether PFF dosage impacts response differs depending on the neurotransmitter^66,67^. The second method used a square excitation waveform frequency of 2000 ms and 1200 mV excitation for 1000 ms for electrical stimulation based on previous literature, with a dopamine dosage applied immediately following^54,55^. MEA recordings were preprocessed in MC_Rack (Multi-Channel Systems) using a bandpass filter of 200–3000 Hz to reduce noise, artifacts, and local field potentials. The spike detection function was used to identify spiking events at a threshold of − 5 standard deviations.

### Substance P ELISA assay

Spent media was collected, centrifuged at 1000 G for 5 min, and stored at – 80 °C. Substance P was measured using an ELISA kit (cat: ADI-900-018A, Enzo Life Sciences) according to protocols.

### Experimental design and statistical analyses

All experiments were conducted in triplicate, with each figure including information on specific sample size and statistics. Experimental replicates were defined as individual experiments, and technical replicates were defined as the number of culture wells. Statistics were calculated in R version 4.1. using the emmeans, lmer4, lmtest, dplyr, glmmTMB, and DHARMa packages. Additionally, ggplot2, emmeans, viridis, and ggsignif were utilized for creating supplementary plots. Prism was utilized for most figure plots. One- or two-way multilevel models were utilized with multiple comparisons and Tukey p-adjustment on either normal or transformed data. Models were evaluated by log-likelihood ratio, where the simplest model significantly improved over a general linear model (if any) was utilized. For non-normal data that could not be transformed, a Kruskal–Wallis test and Wilcoxon rank-sum multiple comparisons with Benjamini–Hochberg p-value adjustment were used. Finally, a zero-inflated negative binomial generalized multilevel model with Tukey p-adjustment was used to analyze electrophysiology recordings for Fig. 6 and Supplementary Fig. S14 (*p < 0.05, **p < 0.01, ***p < 0.001).

## Supplementary Information

Supplementary Information.

## Data Availability

MATLAB (image analysis) and R Studio (statistical analysis) code and collected data and images are available on Harvard Dataverse (dataverse.harvard.edu/dataverse/ens-pff-pd).
