# Supplementary material for: Aggregation of alpha-synuclein in enteric neurons does not impact function in vitro
Source: Sci Rep. 2022 Dec 23;12:22211. doi: 10.1038/s41598-022-26543-x (PMC9789045; doi:10.1038/s41598-022-26543-x)
Supplement: Supplementary file 1 — Supplementary Information. [file 41598_2022_26543_MOESM1_ESM.pdf]

Supplementary Material

Aggregation of alpha-synuclein in enteric neurons does not impact function in vitro

Adam J. Bindas[1], Kyla N. Nichols[1], Nicole J. Roth[3], Ryan Brady[1], Ryan A. Koppes[1], Abigail N. Koppes[1,2]**

Figure S1:

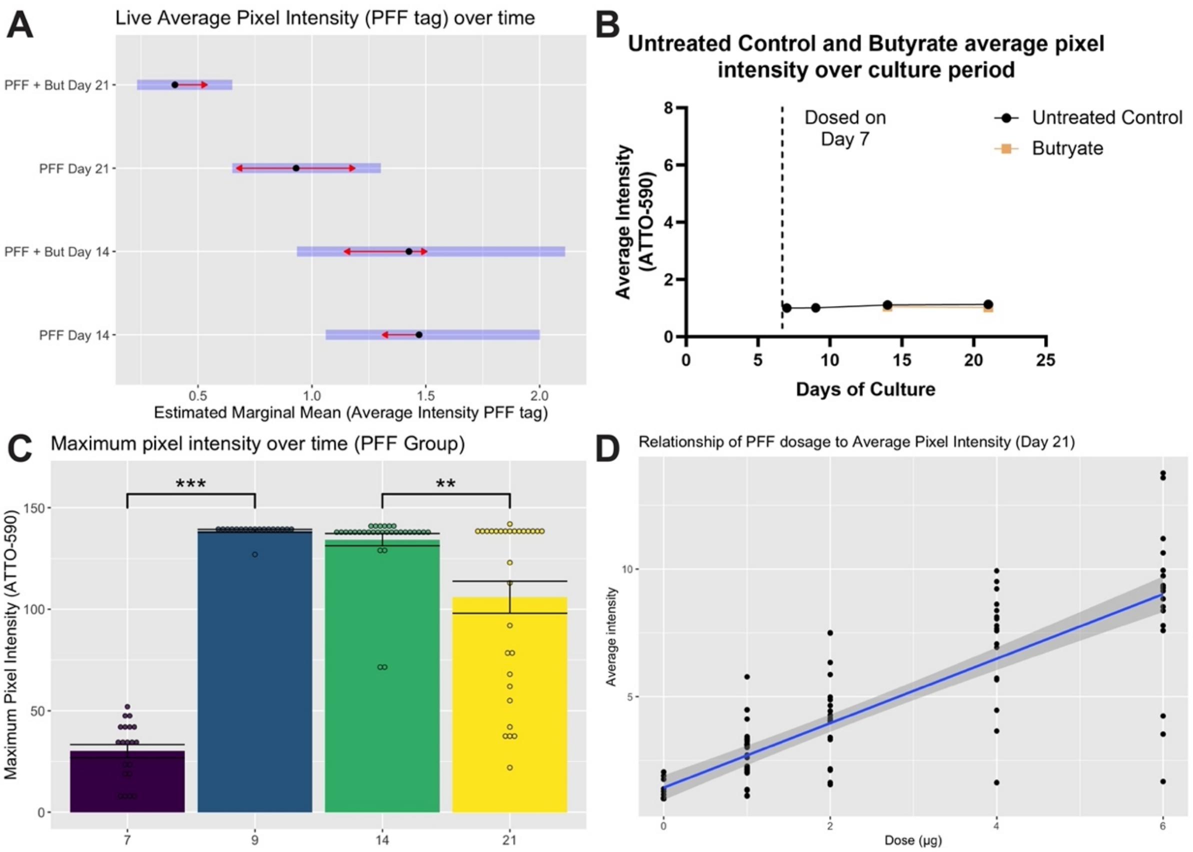

Figure S1 - Additional live-imaging preformed fibril dosage analyses and plots. A) Estimated marginal means plot with significance comparison of longitudinal live-imaging PFF experiment. Box-cox transformed multilevel model adjusting for experimental replicate and Tukey adjusted p-value. Blue box = 95% confidence interval and red arrows = 0.05 p-value comparison arrows. N = 3-6, m = 8-20, images = 10-31. B) Longitudinal plot of collected untreated control and butyrate average PFF tag intensity remains low. N = 3-4, m = 8-12, images = 13-20. C) Plot of maximum intensity for each image within PFF group over time. Kruskal–Wallis test and Wilcoxon rank-sum multiple comparisons with Benjamini-Hochberg p-value adjustment utilized. N = 4-6, m = 12-21, images = 18-31. D) Scatterplot of average pixel intensity on day 21 of culture organized by PFF dosage (μg). Adjusted R^2 = 0.6849, linear model smoothing utilized. N = 4-6, m = 11-20, images = 18-29. Pixel intensity in uint8 (values range from 0-255). Each dot represents an image. ($^{*}p < 0.05$, $^{**}p < 0.01$, $^{***}p < 0.001$)

Figure S2:

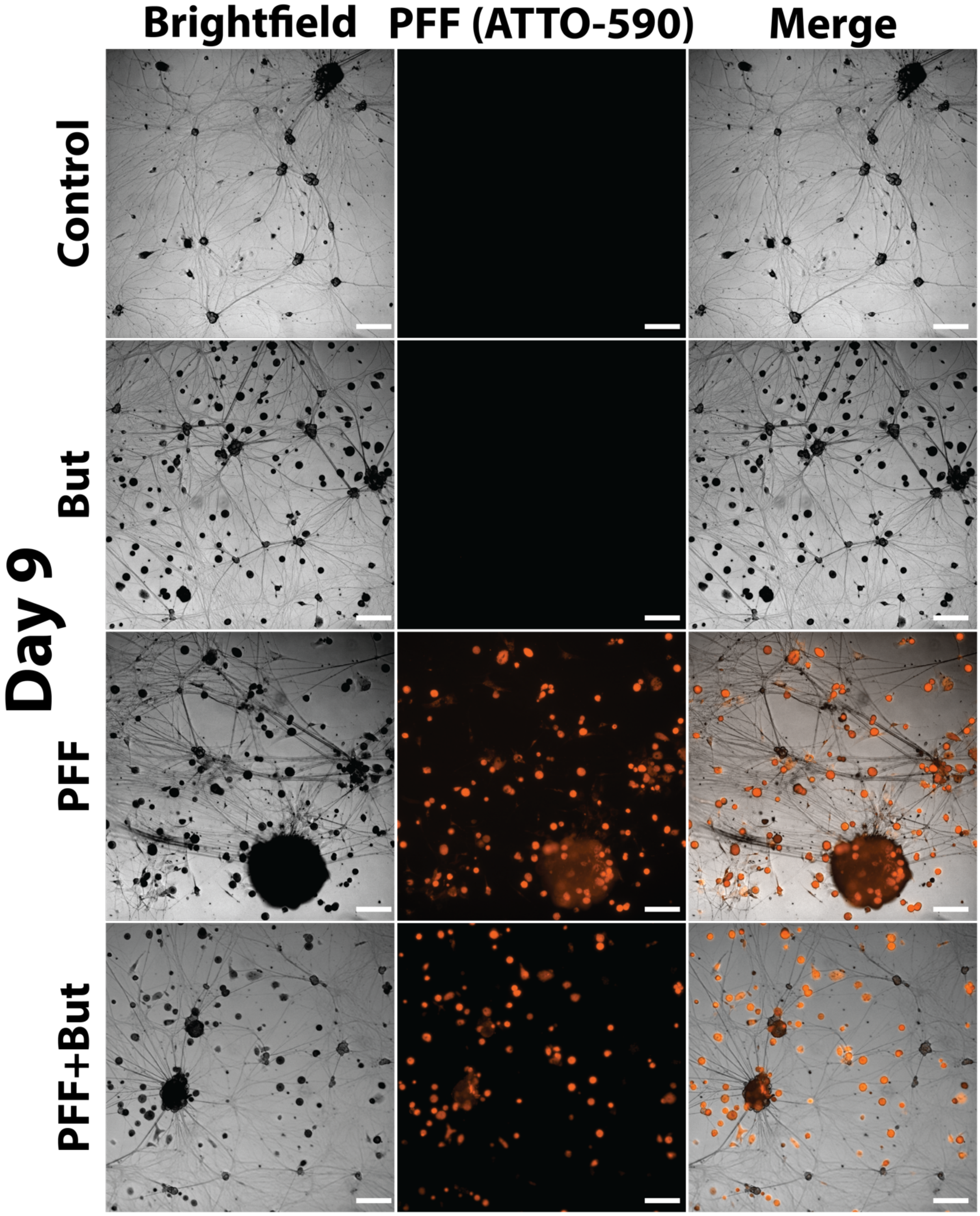

Figure S2 – Representative images of cultures on day 9 between groups. (orange = PFF fluorescent tag). Scale = 100 µm.

Figure S3

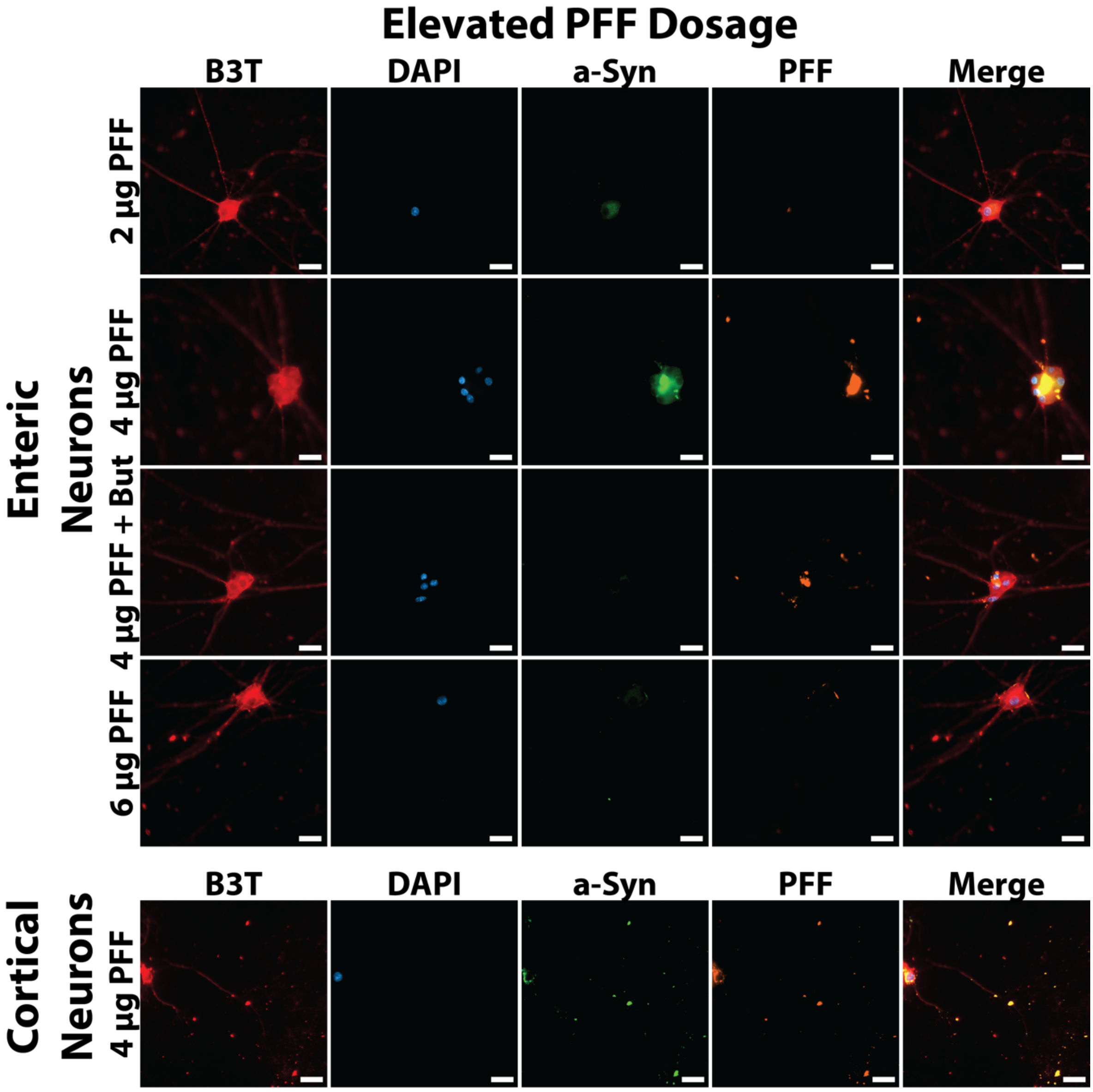

Figure S3 – Additional representative immunostained neural population groups evaluated. (red = beta 3-tubulin (B3T), blue = DAPI, green = alpha-synuclein (a-Syn), orange = preformed fibril fluorescent tag). Overlayed colored boxes indicate neural type and presence of PFFs for experimental groups. Scale = 20 µm.

Figure S4

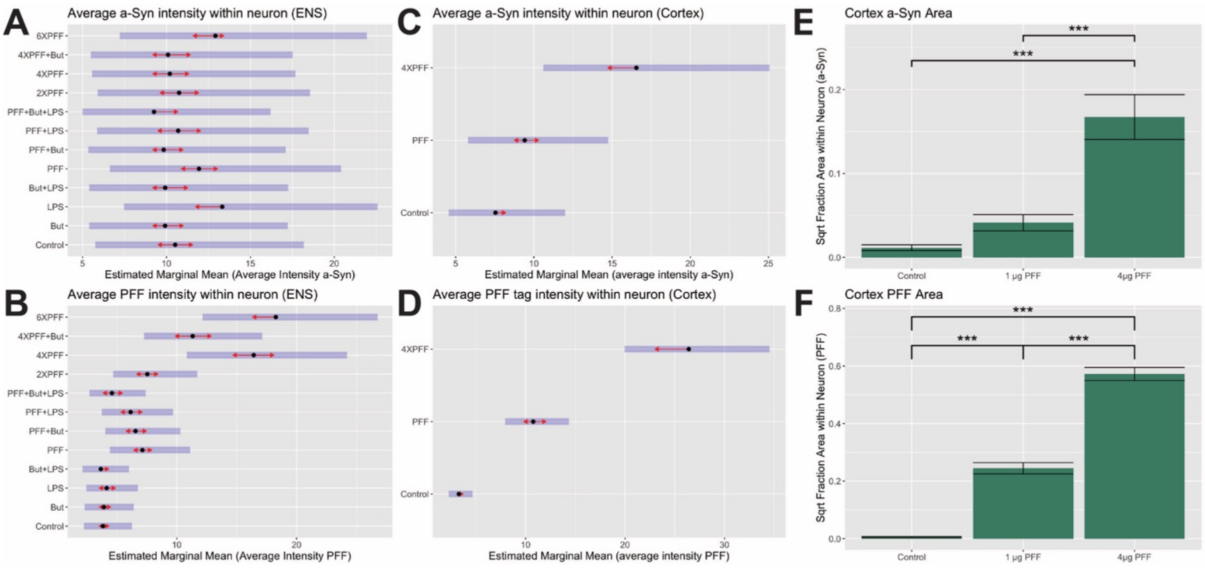

Figure S4 – Estimated means plots of average intensity within neurons and cortical elevated area quantifications. Estimated means plots of average pixel intensity of A-B) ENS and C-D) cortical cultures. Blue box = 95% confidence interval and red arrows = 0.05 p-value comparison arrows. Statistical analysis was conducted using a 6th root transformation multilevel model adjusting for either experimental replicate and well (A) or experimental replicate alone (B-D) with Tukey p-adjustment. E-F) Cortex culture fraction of elevated area for a-Syn and PFF. Statistical analysis was conducted using a Kruskal-Wallis test with a Wilcoxon test for multiple comparisons and Bonferroni p-value adjustment. Pixel intensity in uint8 (values range from 0-255). Error bars = SEM. Sample size ENS: N = 3-7, m = 9-18, images = 60-123. Sample size cortex: N = 4-8, m = 12-27, images = 57-131.

Figure S5

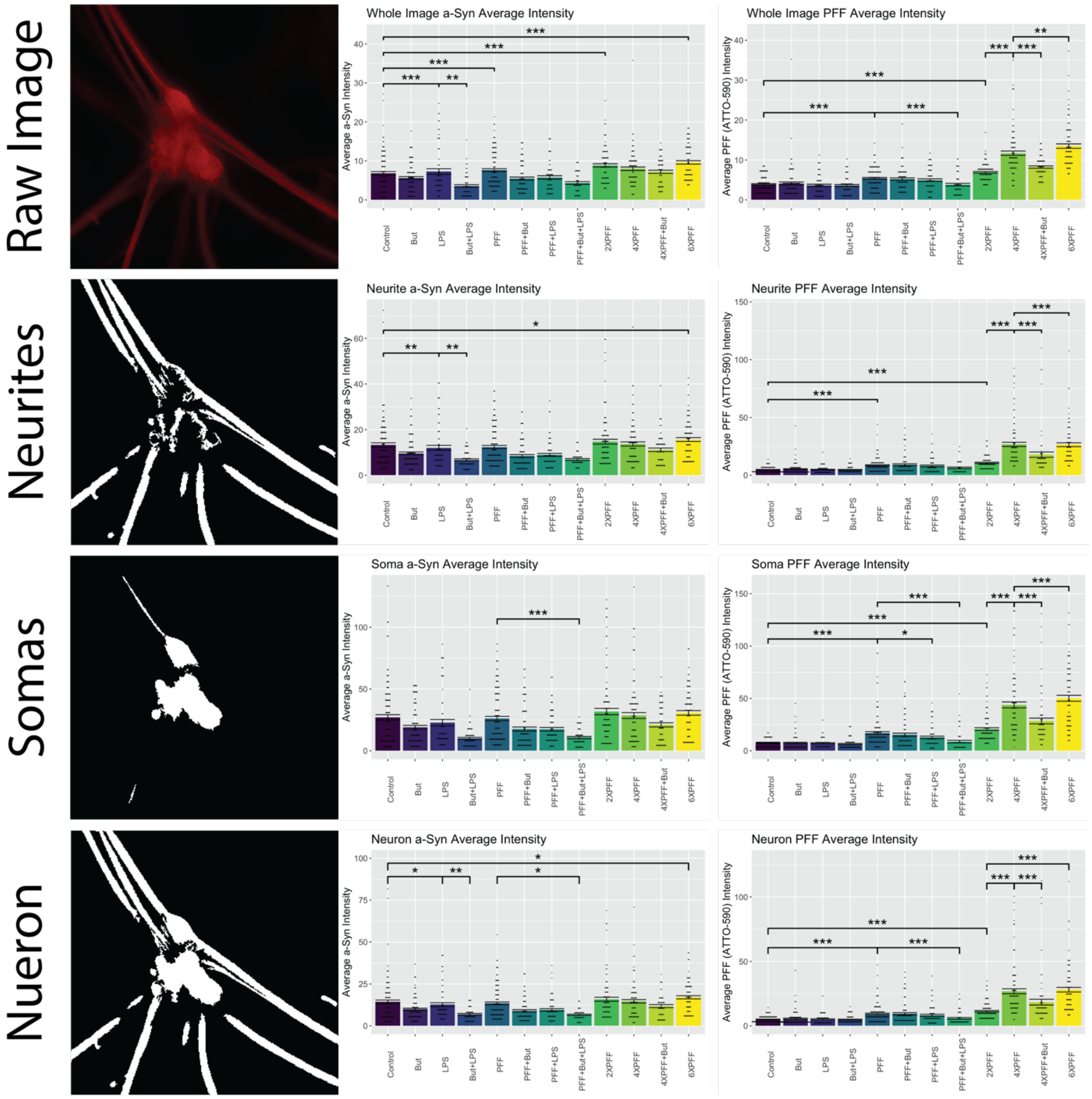

Figure S5 – Breakdown of ENS image average intensity quantification by neural morphology. Organized row-wise (from top to bottom: whole image (no masking), neurites, somas, neurons) with each column representing the same metric (from left to right: a-Syn average intensity, PFF average intensity). Statistical analysis was conducted using a 6th root transformation with a multilevel model adjusting for experimental replicate and well or experimental replicate alone (only neuron morphology PFF) with Tukey p-adjustment. N = 3-7, m = 9-18, images = 60-123. ($^{*}p < 0.05$, $^{**}p < 0.01$, $^{***}p < 0.001$). Pixel intensity in uint8 (values range from 0-255).

Figure S6:

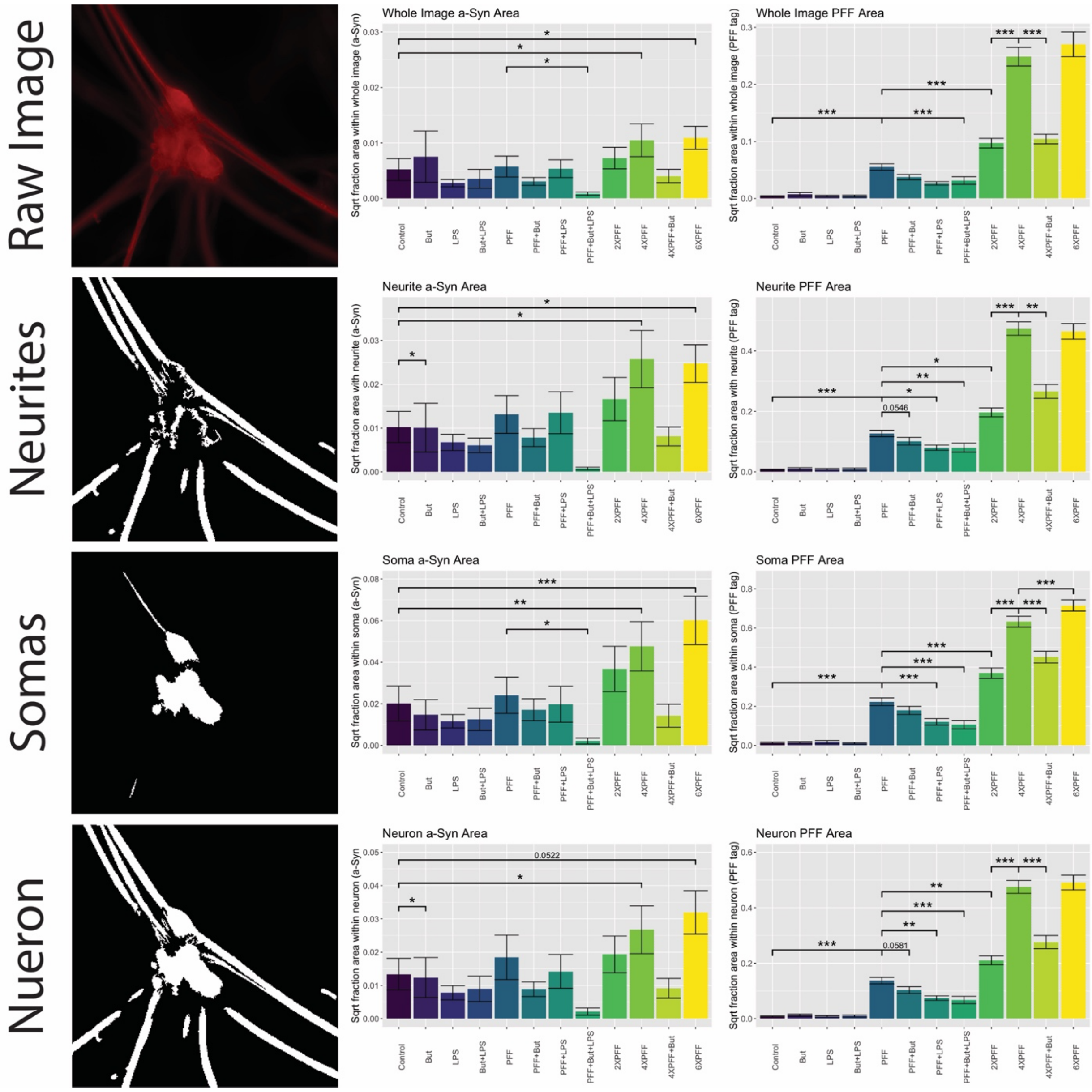

Figure S6 – Breakdown of ENS image quantification of elevated area grouped by neural morphology. Organized row-wise (from top to bottom: whole image (no masking), neurites, somas, neurons) with each column representing the same metric (from left to right: the fraction of elevated a-Syn area, and the fraction of elevated PFF area). Statistical analysis via a Kruskal–Wallis test and Wilcoxon rank-sum multiple comparisons with Benjamini-Hochberg p-value adjustment. N = 3-7, m = 9-18, images = 60-123. ($^{*}p < 0.05$, $^{**}p < 0.01$, $^{***}p < 0.001$). Pixel intensity in uint8 (values range from 0-255).

Figure S7:

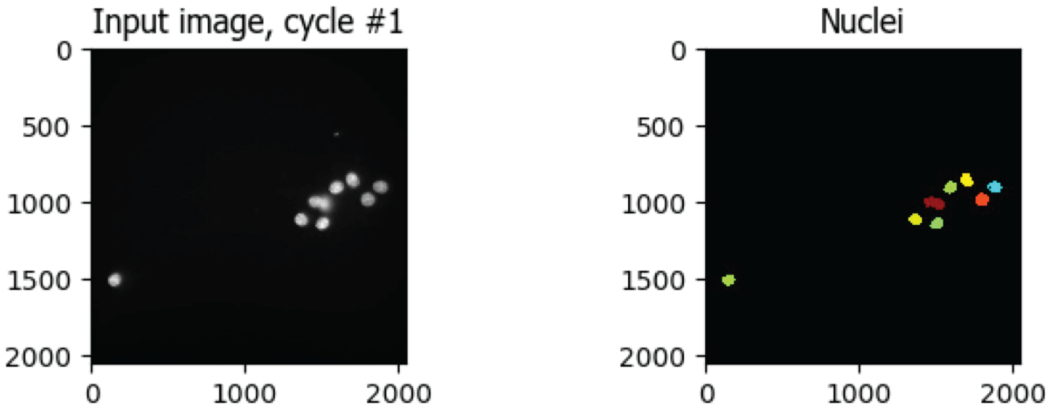

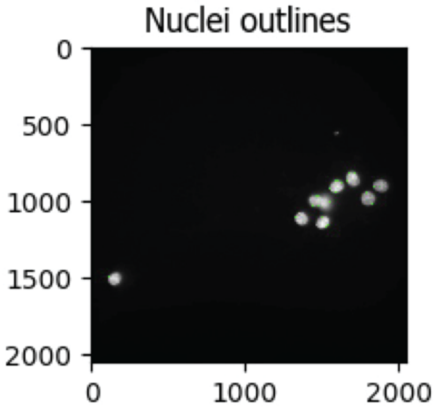

# of accepted objects	8
10th pctile diameter	78.3 pixels
Median diameter	82.2 pixels
90th pctile diameter	103.9 pixels
Area covered by objects	1.1 %
Thresholding filter size	1.0
Threshold	0.199
Declumping smoothing filter size	33.6
Maxima suppression size	35.0

Figure S7 – Representative nuclei identification via CellProfiler. Input DAPI channel image (in grayscale), shown with annotated outlines and colored nuclei areas.

Figure S8:

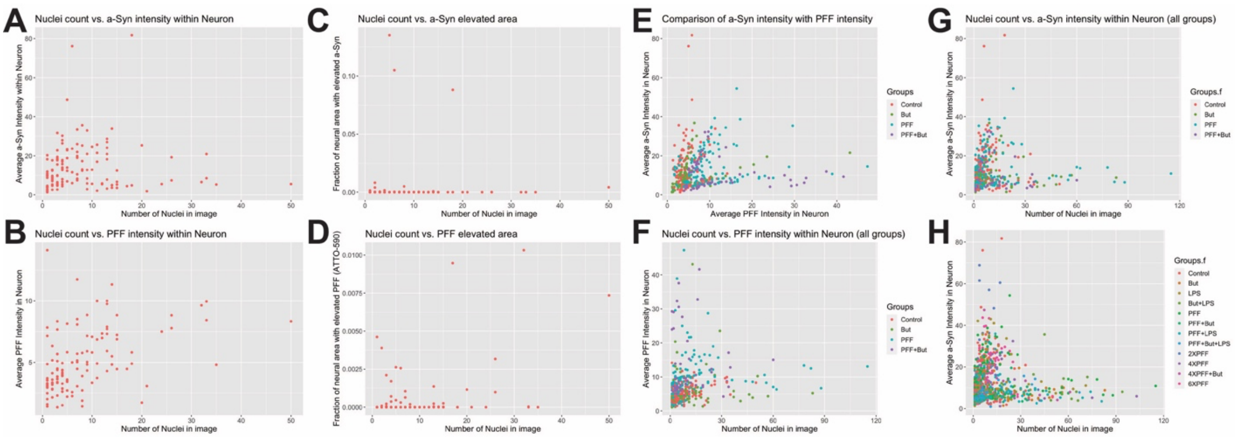

Figure S8 – Scatterplot comparison of average intensity and elevated area by nuclei number for ENS cultures. A-D) Control, or no dosage controls only included, demonstrate no clear relationship between nuclei number and intensity. N = 6, m = 15, images = 69. E-H) including more groups (control, But, PFF, PFF+But), no clear relationships are present between nuclei number and intensity. N = 6, m = 15, images = 37-84. Pixel intensity in uint8 (values range from 0-255). Each dot represents an image.

Figure S9:

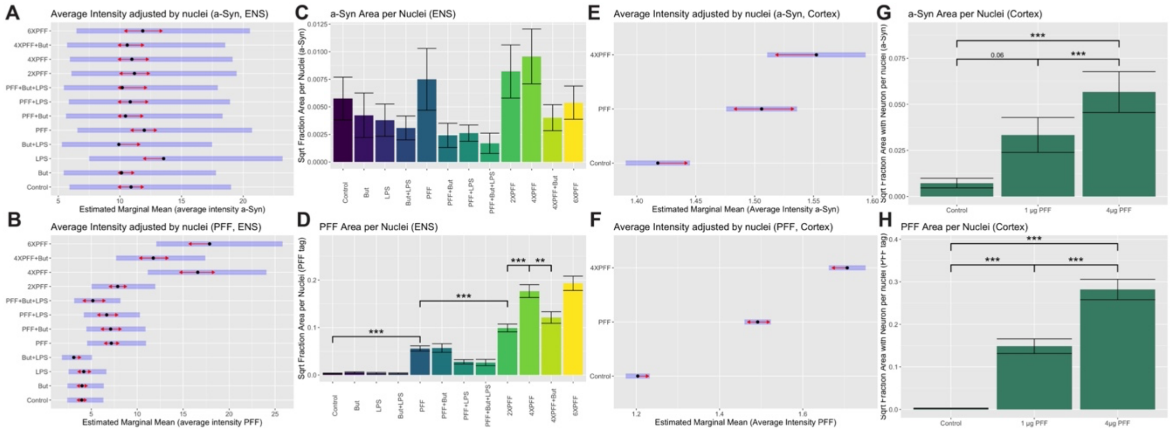

Figure S9 – Nuclei corrected plots corrected for nuclei count. A-B) ENS neurons quantification adjusting for nuclei number. C-D) Elevated area within ENS neuron morphology divided by the number of nuclei. ENS sample size: N = 2-6, m = 5-15, images = 22-69. E-F) Cortical neuron quantification adjusting for nuclei number. G-H) Elevated area within cortical neuron morphology divided by the number of nuclei. Cortex sample size: N = 4-8, m= 12-27, images = 56-128. Blue box = 95% confidence interval and red arrows = 0.05 p-value comparison arrows. Statistical analysis of average intensity results used a 6th root transformation multilevel model adjusting for experimental replicate with Tukey p-adjustment. The analysis of the fraction of elevated area results utilized a Kruskal–Wallis test and Wilcoxon rank-sum multiple comparisons with Benjamini-Hochberg p-value adjustment. Pixel intensity in uint8 (values range from 0-255). (*$p < 0.05$, **$p < 0.01$, ***$p < 0.001$).

Figure S10:

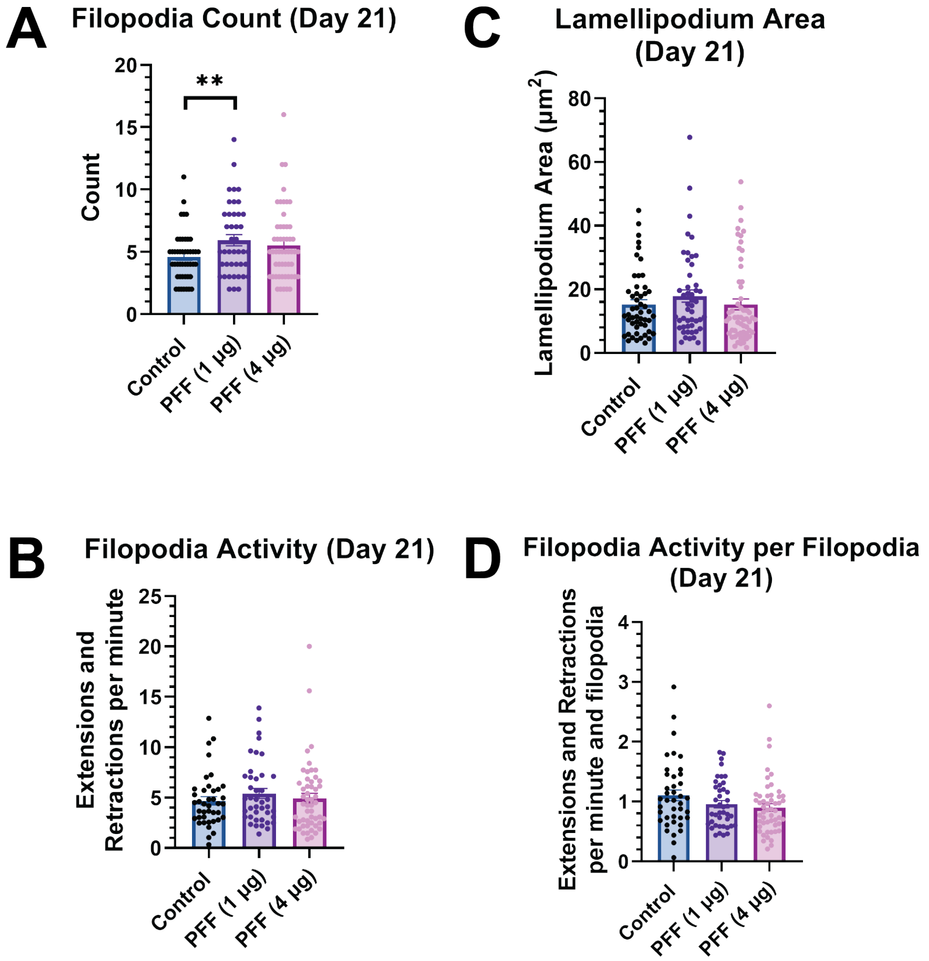

Figure S10 – Growth cone quantitative assessment of ENS cultures observed on day 21 of culture. Day 21 measurement of A) filopodia count, B) filopodial activity (sum of extensions and retraction events per time passed), C) lamellipodium area, D) filopodial activity normalized per filopodia. Statistical analyses conducted with either logarithmic (A,C) or 4th root (B,D) transformation and either general linear model (B-C) or multilevel model adjusting for experimental replicate and well (D) or replicate alone (A). (N = 5, m = 11-13, growth cones = 37-57. Each dot represents a growth cone, error bars = SEM. ($^{*}p < 0.05$, $^{**}p < 0.01$, $^{***}p < 0.001$).

Figure S11:

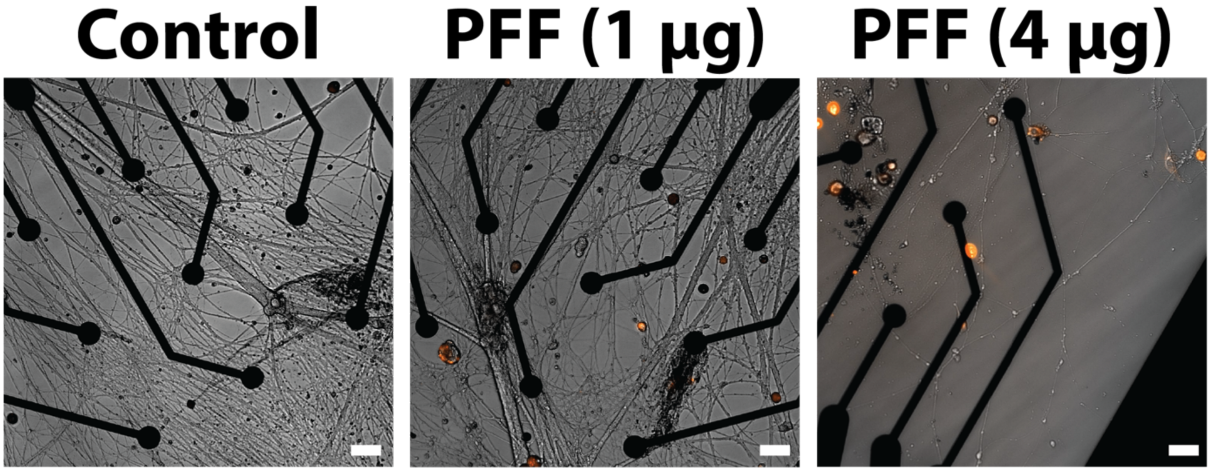

Figure S11 – Representative Images of microelectrode array cultures (orange = PFF tag) on day 28. Scale = 50 µm.

Figure S12:

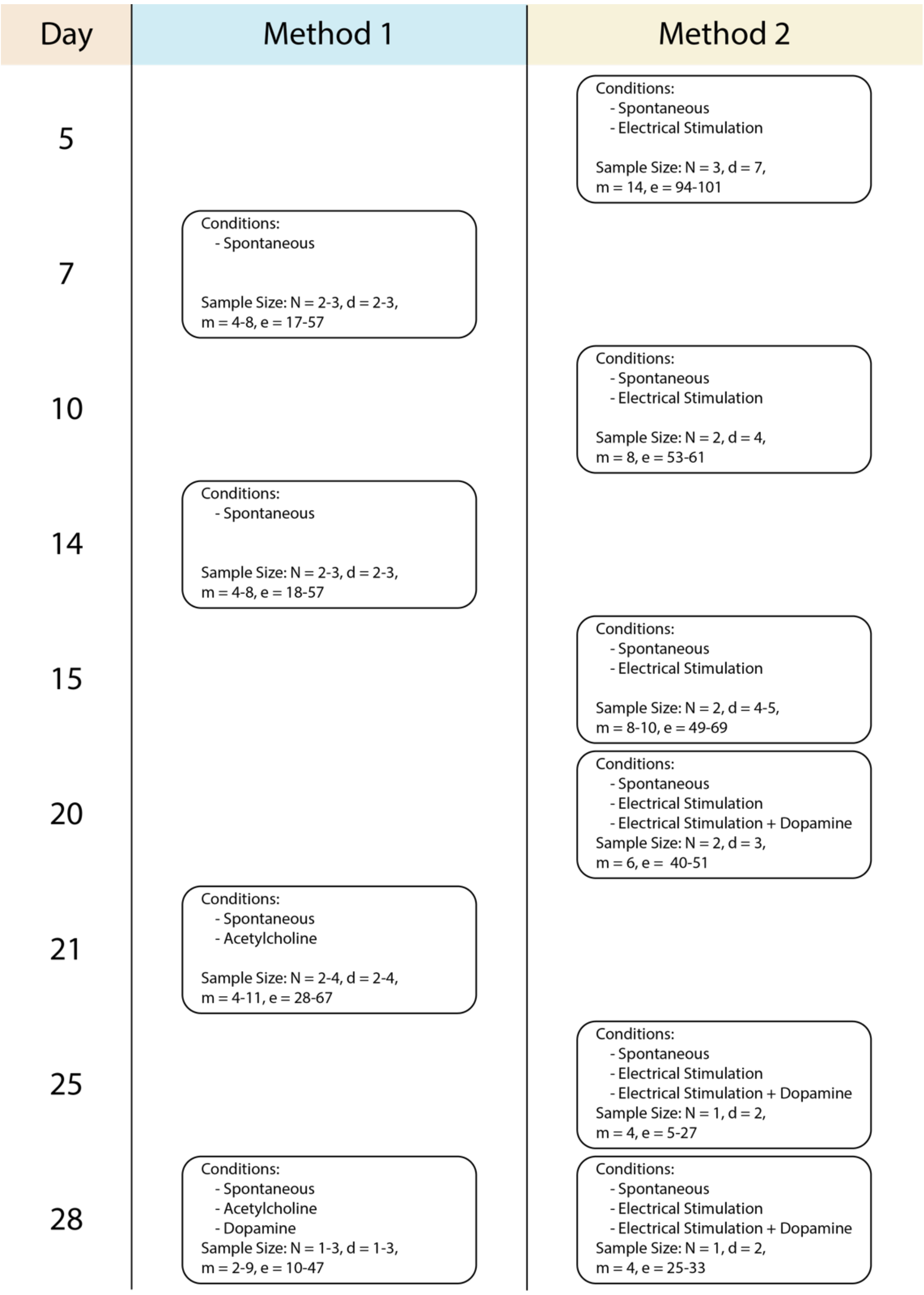

Figure S12 – Experimental methods and sample size diagram for MEA cultures over the culture period. Method 1 utilizes a neurotransmitter-based stimulus, while method 2 uses an electrical stimulation method with dopamine. N = experimental replicates, d = MEA devices, m = wells, e = electrodes.

Figure S13:

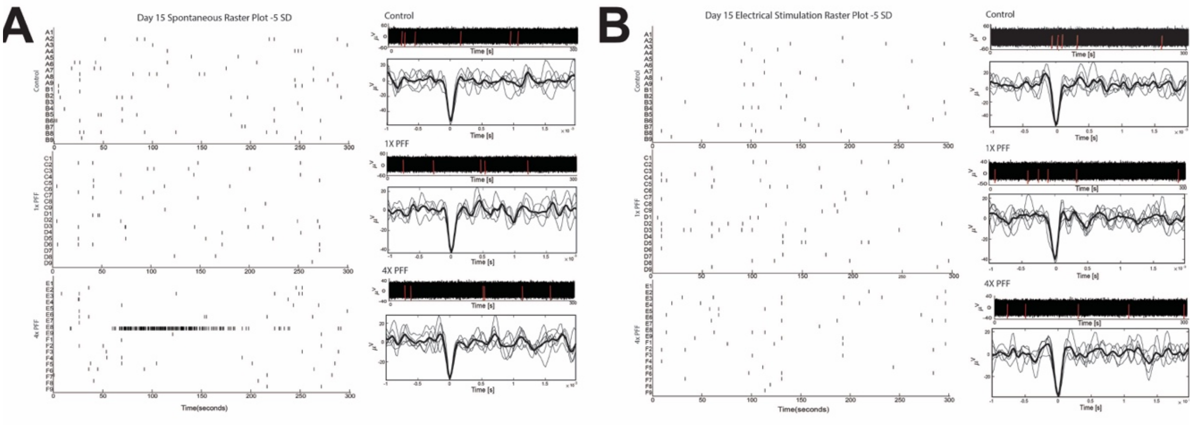

Figure S13 – Representative raster plots, voltage traces, and waveforms. Comparison of A) spontaneous and B) electrically stimulated action potential activity recordings.

Figure S14:

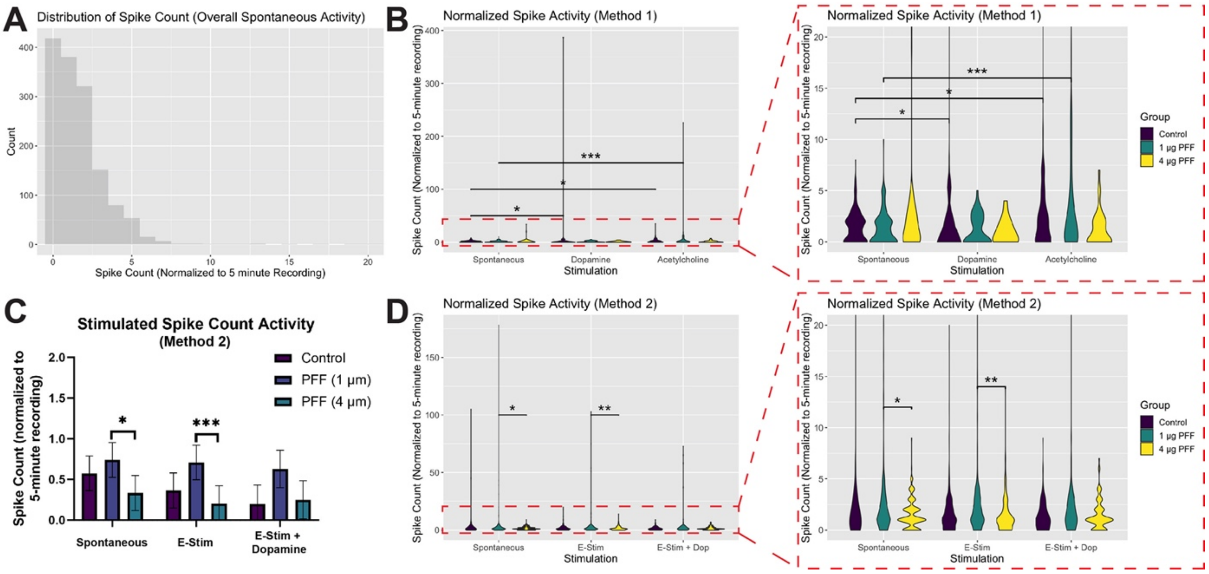

Figure S14 – A) Histogram of overall spontaneous action potential count (spike count) normalized to 5 minutes. Eighteen points (1.2%) were cropped out to illustrate the overall distribution more clearly. B) Violin plot with full and cropped data distribution of method 1 normalized spike count. N = 1-4, number of MEAs = 1-4, wells = 2-11, number of electrodes = 10-50. (N>1 outside day 28, 4 µg PFF. C) Estimated means and standard error of spike count activity of method 2. D) Violin plot with full and cropped data distribution of method 2 normalized spike count. N = 1-2, number of MEAs = 1-5, wells = 2-10, number of electrodes = 18-90. Every sample outside day 28 (4 µg PFF) had more than one replicate. Zero-inflated, negative binomial generalized multilevel models adjusting for culture day and nested random effects for experimental replicate and electrode with Tukey p-adjustment were used for method 1 and 2 analyses. ($^{*}p < 0.05$, $^{**}p < 0.01$, $^{***}p < 0.001$).

Table S1

Group	Day	N	m	# Images
**Control**	7	4	12	20
**PFF**	7	4	12	20
**Control**	9	4	12	19
**PFF**	9	4	12	18
**Control**	14	6	21	31
**But**	14	3	9	15
**PFF**	14	6	21	31
**PFF+But**	14	3	9	15
**Control**	21	6	20	29
**But**	21	3	8	13
**PFF**	21	6	19	29
**PFF+But**	21	3	9	15

Table S1 – Sample size of longitudinal live imaging analyses in **Error! Reference source not found.**. N = experimental replicate, m = Well, # Images = number of images.

Table S2

Group	N	m	# Images
**Control**	6	20	29
**But**	3	8	13
**PFF**	6	19	29
**PFF+But**	3	9	15
**2XPFF**	4	12	19
**4XPFF**	4	11	18
**4XPFF+But**	3	9	14
**6XPFF**	4	12	18

Table S2 - Sample size for day 21 live imaging analysis in **Error! Reference source not found.**. N = experimental replicate, m = Well, # Images = number of images.

Table S3

Group	N	m	# Images
**Control**	7	18	123
**But**	5	`15	106
**LPS**	5	15	72
**But+LPS**	4	12	72
**PFF**	7	19	136
**PFF+But**	5	15	93
**PFF+LPS**	5	14	78
**PFF+But+LPS**	4	12	60
**2XPFF**	4	11	108
**4XPFF**	4	12	104
**4XPFF+But**	3	9	74
**6XPFF**	4	12	98

Table S3 - Sample size for ENS immunostaining quantification experiments in **Error! Reference source not found.**, Figure S4, Figure S5, Figure S6, Figure S7, Figure S8, and Figure S9. N = experimental replicate, m = Well, # Images = number of images.

Table S4

Group	N	m	# Images
**Control**	8	27	131
**PFF**	7	24	118
**4XPFF**	4	12	57

Supplementary Table 4 - Sample size for cortical immunostaining quantification experiments in **Error! Reference source not found.**, Figure S4, and Figure S9. N = experimental replicate, m = Well, # Images = number of images.

Table S5

Group	Day	N	m	# Growth Cones
**Control**	**14**	4	11	54
**Control**	**21**	5	11	48
**PFF**	**14**	4	12	43
**PFF**	**21**	5	12	37
**4XPFF**	**14**	4	12	51
**4XPFF**	**21**	5	13	57

Supplementary Table 5 – Sample size for growth cone analyses in **Error! Reference source not found.** and Figure S10. N = experimental replicate, m = Well, # Images = number of images.

Table S6

Method	Group	Day	Cond.	N	# MEAs	# Wells	# elects.
`2	Control	5	Spont	3	7	14	126
2	PFF	5	Spont	3	7	14	126
2	4XPFF	5	Spont	3	7	14	126
1	Control	7	Spont	3	3	8	72
1	PFF	7	Spont	3	3	8	72
1	4XPFF	7	Spont	2	2	4	36
2	Control	10	Spont	2	4	8	72
2	PFF	10	Spont	2	4	8	72
2	4XPFF	10	Spont	2	4	8	72
1	Control	14	Spont	3	3	8	72
1	PFF	14	Spont	3	3	8	72
1	4XPFF	14	Spont	2	2	4	36
2	Control	15	Spont	2	5	10	90
2	PFF	15	Spont	2	5	10	90
2	4XPFF	15	Spont	2	5	10	90
2	Control	20	Spont	2	3	6	54
2	PFF	20	Spont	2	3	6	54
2	4XPFF	20	Spont	2	3	6	54
1	Control	21	Spont	4	4	11	99
1	PFF	21	Spont	4	4	11	99
1	4XPFF	21	Spont	2	2	4	36
2	Control	25	Spont	1	2	4	36
2	PFF	25	Spont	1	2	4	36
2	4XPFF	25	Spont	1	2	4	36
1	Control	28	Spont	3	3	9	81

**2**	**Control**	**28**	**Spont**	1	2	4	36
**1**	**PFF**	**28**	**Spont**	3	3	9	81
**2**	**PFF**	**28**	**Spont**	1	2	4	36
**1**	**4XPFF**	**28**	**Spont**	1	1	2	18
**2**	**4XPFF**	**28**	**Spont**	1	2	4	36
**1**	**Control**	**29**	**Spont**	1	1	3	27
**1**	**PFF**	**29**	**Spont**	1	1	3	27
**1**	**Control**	**28**	**Dop**	2	2	6	54
**1**	**PFF**	**28**	**Dop**	2	2	6	54
**1**	**4XPFF**	**28**	**Dop**	1	1	2	18
**1**	**Control**	**29**	**Dop**	1	1	3	27
**1**	**PFF**	**29**	**Dop**	1	1	3	27
**1**	**Control**	**21**	**ACh**	2	2	6	54
**1**	**PFF**	**21**	**ACh**	2	2	6	54
**1**	**Control**	**28**	**ACh**	2	2	6	54
**1**	**PFF**	**28**	**ACh**	2	2	6	54
**1**	**4XPFF**	**28**	**ACh**	1	1	2	18
**1**	**Control**	**29**	**ACh**	1	1	3	27
**1**	**PFF**	**29**	**ACh**	1	1	3	27
**2**	**Control**	**5**	**E-Stim**	3	6	12	108
**2**	**PFF**	**5**	**E-Stim**	3	6	12	108
**2**	**4XPFF**	**5**	**E-Stim**	3	6	12	108
**2**	**Control**	**10**	**E-Stim**	2	4	8	72
**2**	**PFF**	**10**	**E-Stim**	2	4	8	72
**2**	**4XPFF**	**10**	**E-Stim**	2	4	8	72
**2**	**Control**	**15**	**E-Stim**	2	4	8	72
**2**	**PFF**	**15**	**E-Stim**	2	4	8	72

**2**	**4XPFF**	**15**	**E-Stim**	2	4	8	72
**2**	**Control**	**20**	**E-Stim**	2	3	6	54
**2**	**PFF**	**20**	**E-Stim**	2	3	6	54
**2**	**4XPFF**	**20**	**E-Stim**	2	3	6	54
**2**	**Control**	**25**	**E-Stim**	1	2	4	36
**2**	**PFF**	**25**	**E-Stim**	1	2	4	36
**2**	**4XPFF**	**25**	**E-Stim**	1	2	4	36
**2**	**Control**	**28**	**E-Stim**	1	2	4	36
**2**	**PFF**	**28**	**E-Stim**	1	2	4	36
**2**	**4XPFF**	**28**	**E-Stim**	1	2	4	36

Supplementary Table 6 - Sample size for microelectrode array electrophysiology analyses in **Error! Reference source not found.**, Figure S11, Figure S12, Figure S13, and Figure S14. Cond. = stimulation condition, N = experimental replicate, # MEAs = Number of microelectrode arrays, # Wells = number of wells, # elects. = number of electrodes, Spont = spontaneous activity, ACh = acetylcholine stimulation, Dop = dopamine stimulation, E-Stim = electrical stimulation.

Table S7

**Group**	**N**
**Control**	7
**But**	7
**PFF**	7
**PFF+But**	7
**LPS**	5
**PFF+LPS**	5
**4XPFF**	4
**4XPFF+But**	4

Table S7 – Sample size for substance P ELISA analysis in **Error! Reference source not found.**. N = experimental replicate.
